# 1,2-β-Oligoglucan Phosphorylase from *Listeria innocua*

**DOI:** 10.1371/journal.pone.0092353

**Published:** 2014-03-19

**Authors:** Masahiro Nakajima, Hiroyuki Toyoizumi, Koichi Abe, Hiroyuki Nakai, Hayao Taguchi, Motomitsu Kitaoka

**Affiliations:** 1 Department of Applied Biological Science, Faculty of Science and Technology, Tokyo University of Science, Noda, Chiba, Japan; 2 National Food Research Institute, National Agriculture and Food Research Organization, Tsukuba, Ibaraki, Japan; 3 Graduate School of Science & Technology, Niigata University, Niigata, Niigata, Japan; University of Illinois at Chicago College of Medicine, United States of America

## Abstract

We characterized recombinant Lin1839 protein (Lin1839r) belonging to glycoside hydrolase family 94 from *Listeria innocua*. Lin1839r catalyzed the synthesis of a series of 1,2-β-oligoglucans (Sop_n_: n denotes degree of polymerization) using sophorose (Sop_2_) as the acceptor and α-d-glucose 1-phosphate (Glc1*P*) as the donor. Lin1839r recognized glucose as a very weak acceptor substrate to form polymeric 1,2-β-glucan. The degree of polymerization of the 1,2-β-glucan gradually decreased with long-term incubation to generate a series of Sop_n_s. Kinetic analysis of the phosphorolytic reaction towards sophorotriose revealed that Lin1839r followed a sequential Bi Bi mechanism. The kinetic parameters of the phosphorolysis of sophorotetraose and sophoropentaose were similar to those of sophorotriose, although the enzyme did not exhibit significant phosphorolytic activity on Sop_2_. These results indicate that the Lin1839 protein is a novel inverting phosphorylase that catalyzes reversible phosphorolysis of 1,2-β-glucan with a degree of polymerization of ≥3. We propose 1,2-β-oligoglucan: phosphate α-glucosyltransferase as the systematic name and 1,2-β-oligoglucan phosphorylase as the short name for this Lin1839 protein.

## Introduction

‘Phosphorylase’ is the general term for enzymes that reversibly phosphorolyze glycosyl linkages to generate sugar 1-phosphates [1], [2]. Phosphorylases are generally thought to be involved in metabolism of specific oligosaccharides or storage polysaccharides in cytosol through their phosphorolytic activity. Reverse phosphorolysis is useful for preparation of specific oligosaccharides because of their strict regioselectivity [1]–[3].

This reversibility makes it possible to practically produce several oligosaccharides from abundantly available natural resources, such as sucrose and starch, by combining the reactions of two phosphorylases. Based on these features, practical preparative methods of several oligosaccharides and polysaccharides have been developed [2], [4]–[8]. These combinations enable practical production of such compounds without using expensive sugar 1-phosphates. However, the number of phosphorylases based on EC numbers is only 19, which is much smaller than those of glycoside hydrolases, and limits the usage of the enzyme class. Therefore, it would be beneficial to find new phosphorylases showing unreported substrate specificities and regioselectivities.

Phosphorylases belong to one of the glycoside hydrolase families (GH) 13, 65, 94, 112, and 130 or the glycosyl transferase families (GT) 4 and 35 on the Carbohydrate-Active EnZymes database (http://www.cazy.org) on the basis of the sequence similarity [9]. Among them, activities of GH94 members reported are cellobiose phosphorylase (EC 2.4.1.20) [10], *N*,*N′*-diacetylchitobiose phosphorylase (EC 2.4.1.280) [11], laminaribiose phosphorylase (EC 2.4.1.31) [12], [13], cellodextrin phosphorylase (EC 2.4.1.49) [14]–[16], cellobionic acid phosphorylase (EC 2.4.1.x) [17] and C-terminal domain of cyclic 1,2-β-glucan synthase (EC 2.4.1.x, CGS) possessing phosphorolytic activity on protein-bound 1,2-β-oligoglucan [18].

In the phylogenetic tree analysis of GH94, enzymes catalyzing the same reactions appeared in the same cluster, except for cellodextrin phosphorylase. We noticed that strains of genus *Listeria* generally possess a gene encoding a GH94 protein at a cluster in which no enzyme has been characterized. In this study, we describe the GH94 protein from *Listeria innocua* with phosphorylase activity specific to 1,2-β-oligoglucans that requires a new EC number.

## Materials and Methods

### Sequence Analysis

ClustalW2 (http://www.ebi.ac.uk/Tools/msa/clustalw2/) was used to perform multiple alignments, and MEGA5.1 was used to construct a phylogenetic tree [19].

### Cloning, Expression, and Purification

Genomic DNA of *L. innocua* Clip 11262 (ATCC BAA-680D) was purchased from the American Type Culture Collection (Manassas, VA, USA). The gene encoding the Lin1839 protein (GenBank accession number: CAC97070.1) was amplified by PCR using KOD plus DNA polymerase (Toyobo, Osaka, Japan) and the genomic DNA as a template. Primer pair was forward primer 5′-GTGGATATccaTGgCAATGTTAAAAG-3′ and reverse primer 5′-ATACACAAAACAACCctcGAGACGG-3′ (lower case represents sequences different from original sequence of the genome sequence of *L. innocua* Clip 11262) containing additional NcoI and XhoI sites (underlined), respectively. The amplified *lin1839* gene was inserted into the NcoI and XhoI sites of pET28a(+) (Novagen, Madison, WI, USA) to encode a His_6_–tag fusion at the C terminus. The constructed plasmid was used to transform *Escherichia coli* BL21 (DE3). The transformant was grown in 1 l of Luria–Bertani medium containing 30 μg/ml kanamycin at 37°C until the absorbance reached 0.8 at 660 nm, followed by induction using 0.1 mM IPTG at 20°C overnight. The cells were collected by centrifugation at 3,900×*g* for 5 min and then suspended in 20 mM MOPS–NaOH buffer (pH 7.5) containing 250 mM NaCl (buffer A). The suspended cells were disrupted by sonication and centrifuged at 27,000×*g* for 20 min. The obtained supernatant was loaded onto a HisTrap FF crude column (5 ml; GE Healthcare, Buckinghamshire, UK) equilibrated with buffer A containing 10 mM imidazole using a AKTA Prime Plus chromatography system (GE Healthcare). After unbound components were washed with buffer A containing 10 mM imidazole, a linear gradient of 10–250 mM imidazole in buffer A was used to elute Lin1839r (actually eluted between 25 and 100 mM of imidazole). An Amicon Ultra 30,000 molecular weight cut-off (Millipore, Billerica, MA, USA) was used to change the buffer in the pooled protein solution to 20 mM MOPS–NaOH buffer (pH 7.5). A theoretical extinction coefficient of 175,450 M^−1^cm^−1^, based on the amino acid sequence, was used to determine the protein concentration spectrophotometrically at 280 nm [20]. Finally, 88 mg of recombinant Lin1839 protein (Lin1839r) was obtained from 1 l of culture medium.

### Size-exclusion Chromatography

Lin1839r (1 ml of 2.0 mg/ml solution) was loaded onto Superdex 200 (Hiload 16/60; GE Healthcare) equilibrated with 50 mM MOPS–NaOH (pH 7.0) containing 150 mM NaCl. Ovalbumin (44 kDa), conalbumin (75 kDa), aldolase (158 kDa), ferritin (440 kDa) and thyroglobulin (669 kDa; GE Healthcare) were used as standard proteins. Blue dextran 2000 (2000 kDa; GE Healthcare) was used to determine the void volume of the column.

### Measurement of Phosphorolytic and Synthetic Activity

The phosphorolytic activity was determined by measuring α-glucose 1-phosphate (Glc1*P*) generated in a reaction mixture on the basis of the phosphoglucomutase-glucose 6-phosphate dehydrogenase method [21]. The reaction was performed in a reaction mixture (200 μl) placed in a well of a 96-well microplate (EIA/RIA plate, 96-well half area, Corning, NY, USA) containing appropriate concentrations of each sugar substrate and inorganic phosphate with 5.0 μM α-glucose 1,6-bisphosphate (Sigma–Aldrich, St. Louis, MO, USA), 5.0 IU/ml glucose 6-phosphate dehydrogenase from *Leuconostoc mesenteroides* (Oriental Yeast, Tokyo, Japan), 6.25 IU/ml phosphoglucomutase from rabbit muscle (Sigma–Aldrich), 0.5 mM thio-NAD^+^ (Oriental Yeast), and 25 mM MgCl_2_ in 100 mM MOPS–NaOH buffer (pH 7.5) at 30°C. The concentration of Glc1*P* was calculated by determining absorbance at 400 nm continuously on a Spectramax 190 (Molecular Devices, CA, USA).

The method of Lowry and Lopez [22], as described below, was used to determine the reverse phosphorolytic activity by measuring the amount of inorganic phosphate released in a reaction mixture. In brief, the enzymatic reaction was performed in a reaction mixture (160 μl) containing 10 mM Glc1*P* and an appropriate concentration of each acceptor in 100 mM MOPS–NaOH buffer (pH 7.5) at 30°C. An aliquot (20 μl) was mixed with 160 μl of 0.2 M sodium acetate buffer (pH 4.0) and 20 μl of 1% ammonium molybdate containing 25 mM sulfuric acid to stop the reaction every 3 minutes. Then, 20 μl of 1% ascorbic acid and 0.05% potassium bisulfate were mixed with the samples. The mixtures were incubated at 37°C for 1 h, and the concentrations of phosphate released were quantified by measuring absorbance at 700 nm. One unit of the phosphorolytic and reverse phosphorolytic activities were defined as the amount of the enzyme producing 1 μmol of Glc1*P* and phosphate, respectively, per minute under the above conditions with the concentrations of all substrates at 10 mM.

### Temperature and pH Profile

The effect of pH on the enzymatic activity using 4.0 μg/ml Lin1839r was determined by measuring synthetic activities under the standard conditions described above and by substituting 100 mM MOPS–NaOH buffer (pH 7.5) with various 100 mM buffers. Similarly, the effect of temperature on the enzymatic activity of 4.0 μg/ml Lin1839r was determined by measuring the reverse phosphorolytic activities at various temperatures for 20 min. The thermal and pH stabilities were evaluated by measuring the residual synthetic activity of 10 mM sophorose (Sop_2_) and 10 mM Glc1*P* after incubation of Lin1839r (0.5 mg/ml) at different temperatures in 20 mM MOPS–NaOH buffer (pH 7.5) for 1 h and at different pH at 30°C for 1 h, respectively.

### Thin Layer Chromatography (TLC)

An aliquot (1 μl) of the reaction mixture for the synthetic reaction was spotted onto a TLC plate (Kieselgel 60 F_254_; Merck, Darmstadt, Germany), and a solution of acetonitrile:water (3∶1, v/v) was used to elute the samples. The TLC plate was soaked in 5% sulfuric acid:methanol solution and heated in an oven until bands were sufficiently visible.

### Structural Analysis of the Reaction Products

Reaction products (oligosaccharides) for structural determination were generated by incubation of a reaction mixture (4 ml) containing 20 mM Glc1*P*, 10 mM Sop_2_, and 40 μg/ml Lin1839r in 100 mM MOPS–NaOH (pH 7.0) at 30°C for 2 h. After using Amberlite MB4 (Organo, Tokyo, Japan) to desalt the sample, it was concentrated and loaded onto a Toyopearl HW-40F column (5.0 cm φ×80 cm; Tosoh, Tokyo, Japan), equilibrated with distilled water and eluted at a flow rate of 5.0 ml/min. Fractions containing trisaccharide and tetrasaccharide were collected, followed by lyophilization. Polysaccharide was synthesised by incubation of a reaction mixture (1 ml) containing 200 mM Glc1*P*, 50 mM glucose, and 125 μg/ml Lin1839r in 100 mM MOPS–NaOH (pH 7.0) at 30°C for 4 days. The products were separated on the same column as described above. Fractions containing polysaccharide were collected and desalted using Amberlite MB4, followed by lyophilization. The amounts of trisaccharide, tetrasacchride, and polysaccharide obtained were 2.3, 1.6 and 7.5 mg, respectively. One-dimensional (^1^H and ^13^C) and two-dimensional [double-quantum-filtered correlation spectroscopy (DQF-COSY), totally correlated spectroscopy (TOCSY), heteronuclear single-quantum coherence (HSQC), and heteronuclear multiple-bond correlation (HMBC)] NMR spectra of the product were acquired in D_2_O with 2-methyl-2-propanol as an internal standard on a Bruker Avance 500 or Bruker Avance 800 spectrometer (Bruker Biospin, Rheinstetten, Germany). Proton signals were assigned on the basis of the DQF-COSY and TOCSY spectra. ^13^C signals were assigned on the HSQC spectra on the basis of the assignment of proton signals. The linkage position in the oligosaccharides was determined by detecting inter-ring cross peaks in each HMBC spectrum.

### Preparation of 1,2-β-oligoglucans and 1,2-β-glucan for Assay

1,2-β-Oligoglucans [Sop_n_, n denotes the degree of polymerization (DP) of the oligosaccharide] was synthesized without using expensive Sop_2_ by incubation of a reaction mixture (20 ml) containing 500 mM Glc1*P*, 250 mM glucose, and 2.0 mg/ml Lin1839r (pH 7.0 adjusted with HCl) at 30°C for 14 days. After performing electrodialysis on a Microacylizer S1 with an AC-220-10 cartridge (Astom Corp., Tokyo Japan) to remove the salts contained in the reaction mixture, a Toyopearl HW-40F column, as described above, was used to separate the products. Fractions containing Sop_3_, Sop_4_, and Sop_5_ were collected, followed by lyophilization. 1,2-β-glucan was produced by incubation of a reaction mixture (50 ml) containing 750 mM Glc1*P*, 180 mM glucose, and 80 μg/ml Lin1839r in 375 mM MOPS–NaOH (pH 7.0) at 30°C for 8 days. The product was separated as described above. The collected sample solution was concentrated into approximately 15 ml, and then an equal volume of ethanol was added to the 1,2-β-glucan solution. The first precipitate was separated from the supernatant by centrifugation after incubation of the sample at 20°C for 1 day. The second precipitate generated in the first supernatant after the incubation for an additional 1 day was also collected by centrifugation. The final precipitate was obtained after storage of the second supernatant at −30°C for over 1 year. The precipitates were dried under vacuum. The average DP of 1,2-β-glucan was calculated from the ratio of the peak area of C-6 protons of the internal glucose units appearing near 3.94 ppm against that of the C-2 proton at the non-reducing end appearing near 3.33 ppm.

### Kinetic Analysis

The initial velocities of the phosphorolytic reactions with Sop_n_s were determined under the standard conditions with 1.0 μg/ml Lin1839r and a combination of initial concentrations of each substrate and Pi. The kinetic parameters for Sop_3_ were calculated by curve-fitting the experimental data to the theoretical equation (1) for a sequential Bi Bi mechanism using GraFit version 7.0.3 (Erithacus Software Ltd., London, UK).
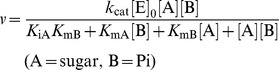
(1)


Kinetic analysis of the synthetic reactions with suitable acceptors was performed under the standard conditions with 2.0 μg/ml Lin1839r and different concentrations of the acceptor substrate or Glc1*P* as the donor with 10 mM each opposite substrate. The kinetic parameters were calculated by curve fitting the experimental data to the Michaelis-Menten equation (2) using GraFit version 7.0.3.
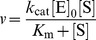
(2)


## Results

### Sequence Analysis

**Figure 1:**
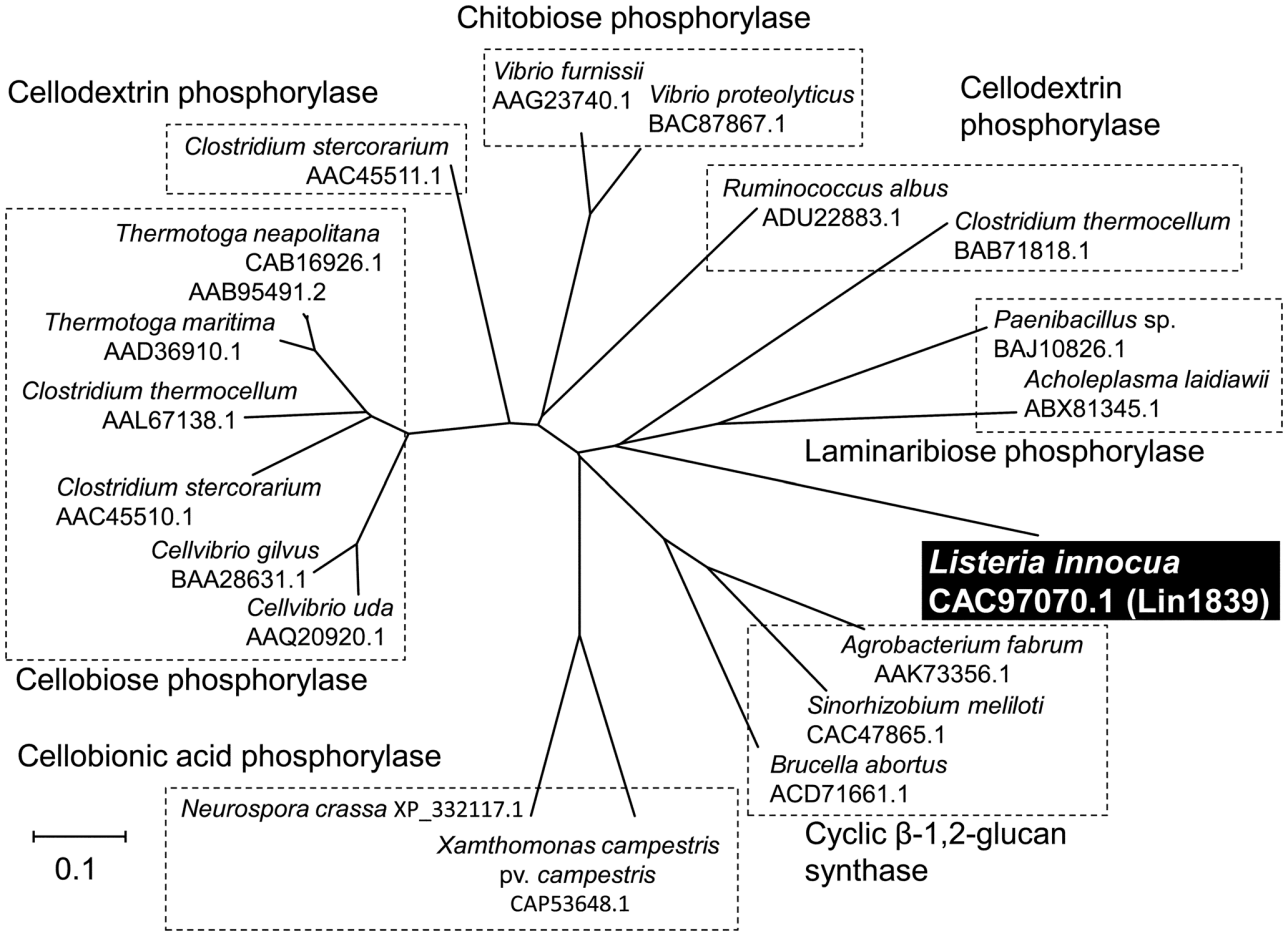
Phylogenetic tree of GH94 proteins. Genes are represented with the organism names and GenBank™ accession numbers. Characterized enzymes are categorized in boxes framed with broken lines according to their substrate specificities. In the case of CGSs, only GH94 domains are used for the alignment. The gene cloned in this study is represented with a black background and white letters. GH94 proteins from *Listeria* are boxed with a bold line.

*L. innocua* Clip11262 possesses a single gene encoding the GH94 protein (Lin1839) in its genome (GenBank accession number: AL592022.1). Alignment of the amino acid sequences of GH94 proteins revealed that the common catalytic nucleophile aspartate residue was conserved as D739 in Lin1839. The amino acid sequence of the Lin1839 protein showed no predicted N-terminal signal peptide on the basis of a SignalP 4.0 analysis (http://www.cbs.dtu.dk/services/SignalP/) [23], suggesting that it is a cytosolic protein. Its activity was not predictable from the phylogenetic tree analysis with other characterized GH94 enzymes (Fig. 1).

### Substrate Specificity in the Synthetic Reaction

**Figure 2:**
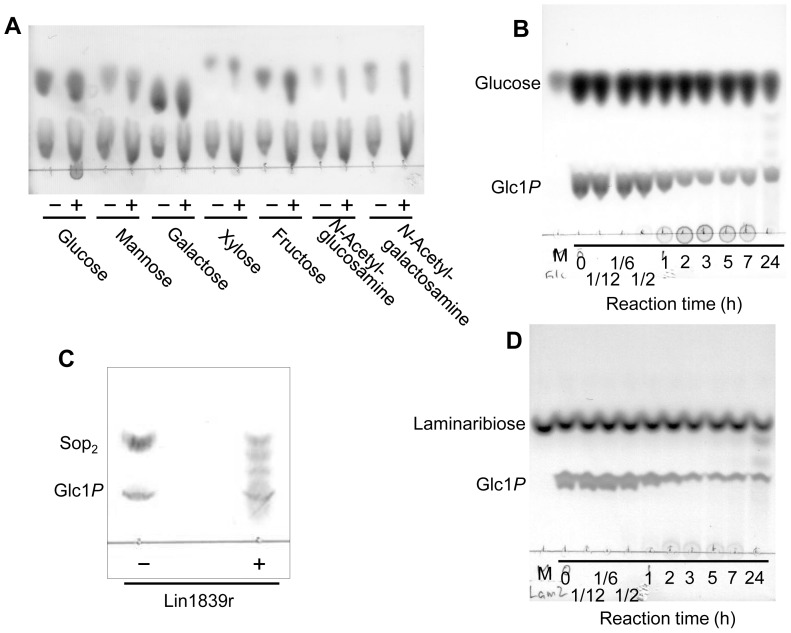
TLC analysis of reaction products from acceptors and Glc1*P*. (A) Reaction products from monosaccharides and Glc1*P*. Reactions were performed with 2 mg/ml of Lin1839r in the presence of 50 mM monosaccharides and 50 mM Glc1*P* for 1 h. (B) Time course of the reaction products from glucose and Glc1*P*. Substrates used were 50 mM glucose and 50 mM Glc1*P*. The enzyme concentration used was 2 mg/ml. (C) Reaction products after reaction for 2 h using 10 mM Sop_2_ and 20 mM Glc1*P* as substrates. (D) Time course of the reaction products from laminaribiose and Glc1*P*. The substrates used were 10 mM laminaribiose and 10 mM Glc1*P*. The enzyme concentration used was 0.5 mg/ml. (A, C) Presence and absence of Lin1839r are represented with ‘+’ and ‘−’, respectively. (B, D) M, marker. Numbers under a line represent reaction time.

The acceptor specificity of Lin1839r in the synthetic reaction was examined using 10 mM of various putative carbohydrate acceptors in the presence of 10 mM Glc1*P* as the donor. Lin1839r did not utilize any monosaccharides as the acceptor to significantly liberate inorganic phosphate under the reaction conditions described in Table 1. We noticed that a spot of probable polysaccharide appeared at the origin of the TLC for the reactions with 50 mM monosaccharides and 50 mM Glc1*P* with higher concentrations of Lin1839r (2 mg/ml) only when glucose was used as the acceptor, but no oligosaccharides were detected (Fig. 2A). After a long incubation period, a series of oligosaccharides were detected on TLC (Fig. 2B). Next, we examined the acceptor specificity for disaccharides at 10 mM. The enzyme showed the highest activity with Sop_2_ (43 s^−1^) and a detectable activity with laminaribiose (0.48 s^−1^) but no activity with other disaccharides (Table 1). The reaction with Sop_2_ produced a series of oligosaccharides (Fig. 2C). The reaction with laminaribiose as the acceptor produced a polymer initially followed by generation of oligomers (Fig. 2D), a pattern similar to that for glucose. The enzyme exhibited activity only on Glc1*P* among the sugar 1-phosphates examined (Table 1).

**Table 1:** Substrate specificity of Lin1839r in the synthetic reaction.

Substrate	Relative activity^a^ (%)
Acceptor^b^	
d-Glucose	N.D^d^^,^ e
2-Deoxy-d-glucose	N.D
d-Xylose	N.D
d-Mannose	N.D
d-Galactose	N.D
l-Fucose	N.D
l-Arabinose	N.D
l-Rhammose	N.D
d-Glucosamine	N.D
*N*-Acetyl-d-glucosamine	N.D
d-Galactosamine	N.D
*N*-Acetyl-d-galactosamine	N.D
Sucrose	N.D
Maltose	N.D
Lactose	N.D
Sophorose	100 (43^f^)
Laminaribiose	1.1 (0.48)
Cellobiose	N.D
Gentiobiose	N.D
Donor^c^	
Glc1*P*	100 (43)
α-d-Galactose 1-phoshate	N.D
α-d-Mannose 1-phoshate	N.D
*N*-Acetyl-α-d-glucosamine 1-phoshate	N.D

a The specific activity of Lin1839r in the presence of 10 mM Sop_2_ and 10 mM Glc1*P* was defined as 100% relative activity.

b Measured at 10 mM with 10 mM Glc1*P* as the donor.

c Measured at 10 mM with 10 mM Sop_2_ as the acceptor.

d N.D represents not determined owing to <0.2% relative activity.

e The specific activity on glucose at 100 mM was 0.095 s^−1^.

f Values in parentheses represent specific activity (s^−1^).

### Basic Properties of Lin1839r

**Figure 3:**
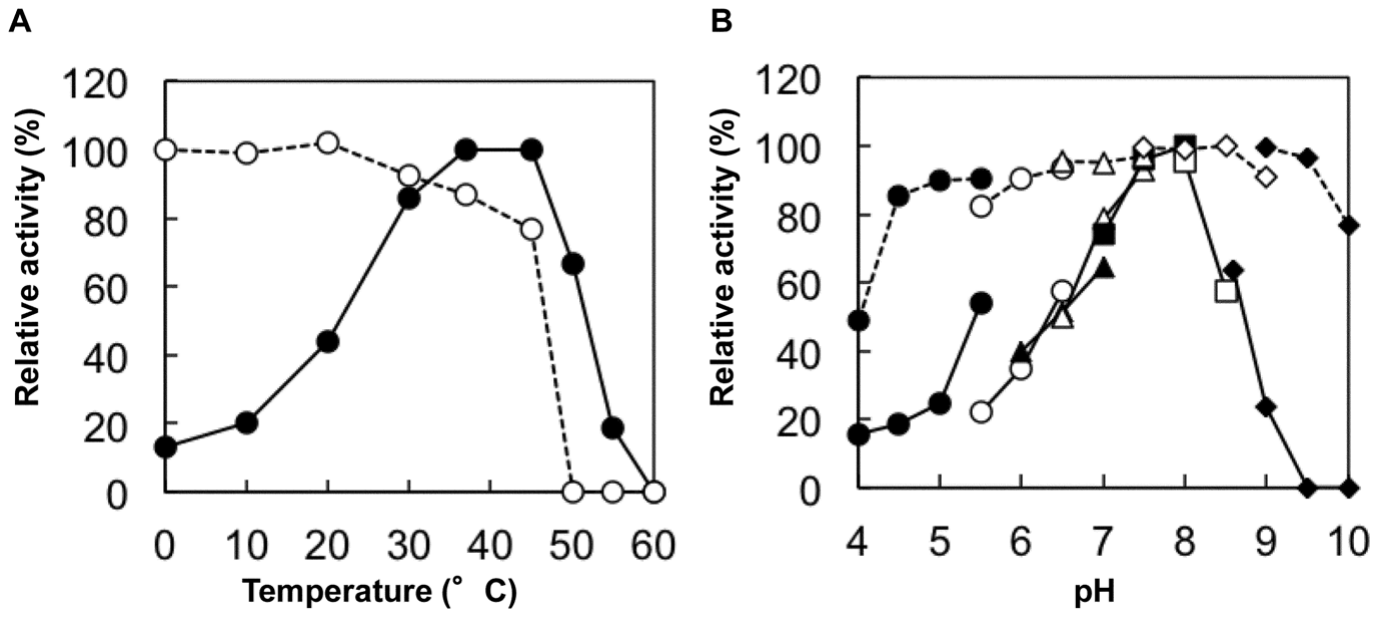
Effects of temperature and pH on enzyme activity and stability of Lin1839r. (A) The temperature optimum and stability are represented by closed and open circles, respectively. (B) The optimum pH and stability are represented by a single line and a dashed line, respectively. The buffers used for reaction and incubation are sodium acetate (pH 4.0–5.5, closed circle), MES–NaOH (pH 5.5–6.5, open circle), BISTRIS (pH 6.0–7.0, closed triangle), MOPS–NaOH (pH 6.5–7.5, open triangle), HEPES–NaOH (pH 7.5–8.5, closed square), 2-hydroxy-3-[4-(2-hydroxyethyl)-1-piperazinyl]propanesulphonic acid–NaOH (pH 8.0–8.5, open square), Tris–HCl (pH 7.5–9.0, open rhombic), and glycine–NaOH (pH 8.6–10, closed rhombic).

Purified Lin1839r was detected at 120 kDa as a single band on SDS-PAGE, which corresponded to the theoretical molecular mass of Lin1839r with His_6_ tag (123,817 Da). Size-exclusion chromatography of Lin1839r resulted in elution of Lin1839r as a 110-kDa protein, suggesting that Lin1839r is a monomeric protein. Lin1839r was stable up to 37°C. The remaining activity of Lin1839r after incubation for 20 min drastically decreased at temperatures >45°C. The optimal temperature was 37–45°C (Fig. 3A). Lin1839r was stable in the range of pH 4.5–9.5, and its optimal pH was 7.5–8.0 (Fig. 3B).

### Analysis of Products of the Synthetic Reaction Catalyzed by Lin1839r

Each oligosaccharide produced in the reaction mixture with Sop_2_ as the acceptor and Glc1*P* as the donor was isolated by gel filtration. The trisaccharide and tetrasaccharide produced were identified as Sop_3_ and Sop_4_, respectively, on the basis of the NMR spectra (Fig. S1–S2, Table S1–2). The polymer product produced from glucose and Glc1*P* at the early stage of the reaction was also isolated by gel filtration. Simple ^1^H and ^13^C NMR spectra of the compound indicated that it is a homopolymer containing only a 1,2-β-glucosyl linkage and an average DP of 39 (Fig. S3, Table S3).

### Preparation of 1,2-β-glucan and Sop_n_s

High concentrations of glucose and Glc1*P* as substrates were successfully adapted to production of 1,2-β-glucan (at a low concentration of Lin1839r) and Sop_n_s (at a high concentration of Lin1839r for a long reaction term) at a larger scale. No precipitate was generated during the entire reaction period, suggesting that the generated 1,2-β-glucan was very soluble in water. For the production of the polymer, addition of ethanol to the 1,2-β-glucan solution caused gradual precipitation. The amounts of the first, second and third precipitates were 1.3, 0.90 and 0.25 g, respectively. The average DPs of these precipitates were 77, 64 and 39, respectively. The amounts of Sop_2_, Sop_3_, Sop_4_ and Sop_5_ obtained were 98, 120, 100 and 35 mg, respectively.

### Kinetic Analysis of the Reverse and Forward Phosphorolytic Reactions

In the reverse phosphorolytic reaction, the kinetic parameters of Lin1839r for activity on the Sop_n_s as acceptors were determined. The enzyme showed similar values of *k*_cat_ and *K*_m_ for Sop_2_, Sop_3_, and Sop_4_, which led to similar *k*_cat_/*K*_m_ values for these oligosaccharides (Table 2). The kinetic parameters for Glc1*P* were in the same range as those of other inverting phosphorylases [13], [24], [25]. In the phosphorolytic reaction, the kinetic parameters of Lin1839r for the Sop_n_s and 1,2-β-glucan (DP = 77) as a substrate were determined. The enzyme exhibited similar *k*_cat_ and *K*_m_ values for Sop_3_, Sop_4_, and Sop_5_ (Table 3). The *k*_cat_/*K*_m_ values for 1,2-β-glucan were much smaller than those for Sop_3_, Sop_4_ and Sop_5_, but the activity was significantly detectable. The enzyme showed only a negligible phosphorolytic activity on Sop_2_ (Table 3). These results indicated that the enzyme catalyzed reversible phosphorolysis of Sop_n_s with ≥DP 3.

**Table 2:** Kinetic parameters for Sop_n_s in the synthetic reaction.

Substrate	*k*_cat_ (s^−1^)	*K*_m_ (mM)	*k*_cat_/*K*_m_ (s^−1^mM^−1^)
Sop_2_ a	97±4	8.5±0.6	11±1
Sop_3_ a	110±10	6.0±0.9	18±1
Sop_4_ a	90±5	6.8±0.7	13±1
Glc1*P* b	43±2	1.2±0.2	34±4

a Up to 10 mM acceptors were used. 10 mM Glc1*P* was used as a donor.

b Up to 10 mM Glc1*P* was used. 10 mM Sop_2_ was used as an acceptor.

**Table 3:** Kinetic parameters for phosphorolysis of Sop_n_s and 1,2-β-glucan.

Substrate	*k*_cat_ (s^−1^)	*K*_m_ (mM)	*k*_cat_/*K*_m_ (s^−1^mM^−1^)
**Sop_2_** a	–c	–	–
**Sop_3_**	19±1	1.0±0.1	19±2
**Sop_4_**	30±1	1.2±0.1	24±1
**Sop_5_**	31±1	1.8±0.1	17±1
**1,2-β-Glucan** b	–	–	0.40±0.07

a Specific activity was <0.2 (s^−1^) at 10 mM.

b Up to 4 mg/ml 1,2-β-glucan (average DP = 77) was used.

c –, not determined.

### Reaction Mechanism of Phosphorolysis

**Figure 4:**
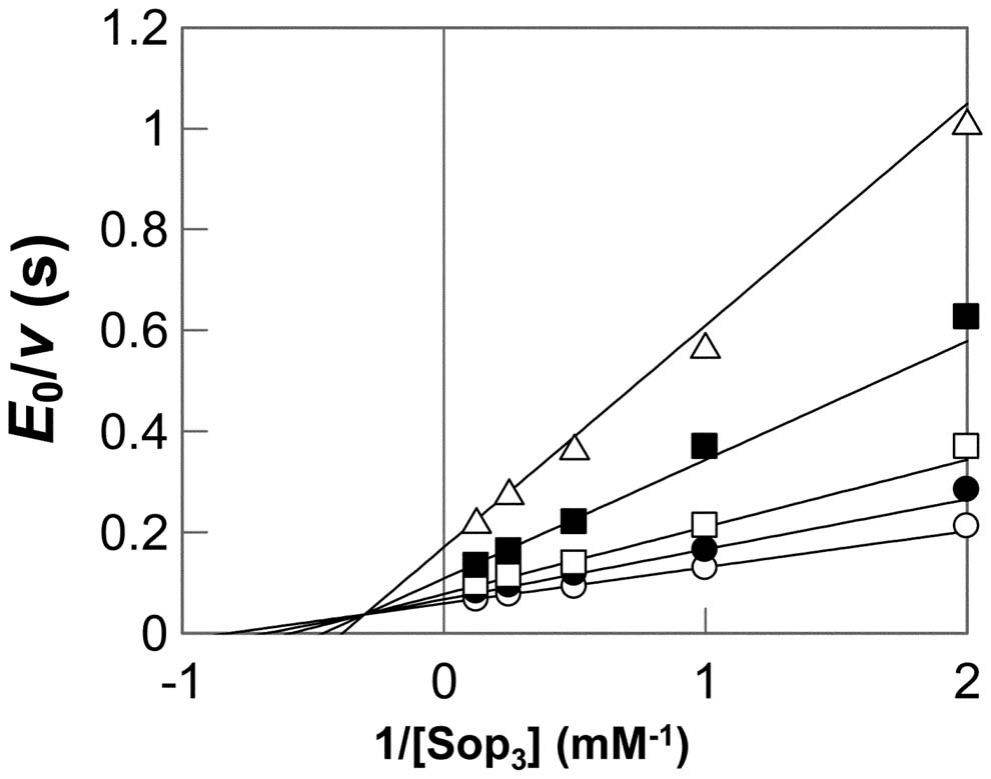
Double reciprocal plot for the phosphorolysis of Sop_3_ with different concentrations of inorganic phosphate. Concentrations of inorganic phosphate were 0.5(open triangle), 1.0 mM (filled square), 2.0 mM (open square), 3.0 mM (filled circle), and 5.0 mM (open circle). The kinetic parameters are as follows: *k*_cat_ = 21±1 (s^−1^), *K*_mA_ = 0.66±0.14 (mM), *K*_mB_ = 1.3±0.2 (mM), and *K*_iA_ = 3.3±0.6 (mM), where A represents Sop_3_ and B is Pi. Grafit version 7.0.3 was used to perform non-linear regression and calculation of values.

Double reciprocal plots of initial velocities against various initial concentrations of Sop_3_ and phosphate yielded a series of lines intersecting at a point (Fig. 4). This result indicated that the phosphorolytic reaction on Sop_3_ followed a sequential Bi Bi mechanism, same as inverting phoshorylases [24], [26]–[28]. The kinetic parameters of Lin1839r determined by regression data are shown in the Fig. 4 legend. The values of *k*_cat_, *K*_mA_, and *K*_mB_ were in the same range as those of other inverting phosphorylases [24], [28]–[30].

## Discussion

### Classification of Lin1839 Protein

**Figure 5:**
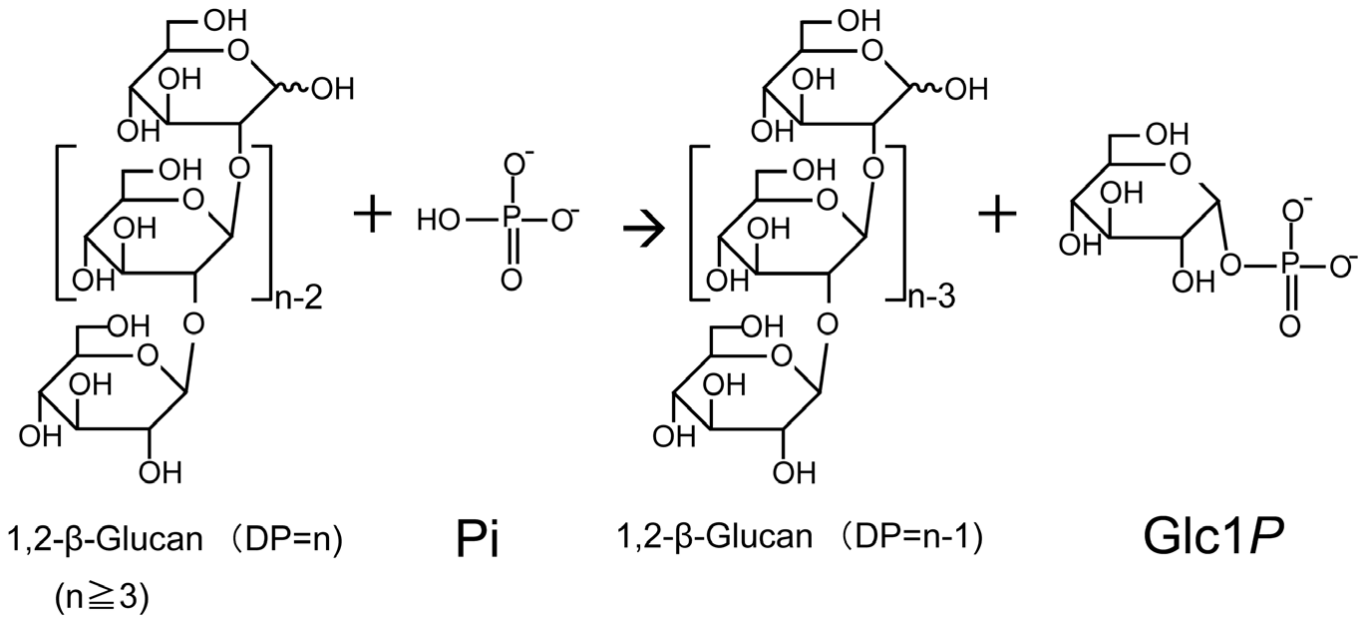
Reaction scheme for the Lin1839 protein.

We found that Lin1839r has phosphorylase activity highly specific to Sop_n_s with ≥DP 3 in phosphorolysis (Fig. 5). The specificity on DP is similar with that of cellodextrin phosphorylase and implies the existence of subsites −1 to +2 in Lin1839 protein. The structural prediction of the architecture of Lin1839 is not possible due to the poor identities (less than 20%) in the amino acid sequence with other GH94 enzymes whose structures have been solved [10], [11], [31], [32].

The C-terminal GH94 domain of CGS from *Brucella abortus* possesses phosphorolytic activity on 1,2-β-glucan [18]. However, Lin1839r is clearly different from the CGS in the catalytic reaction with respect to factors described below.

CGS is a fusion enzyme composed of GT84 and GH94 that synthesizes cyclic 1,2-β-glucan (CβG) [33], [34]. CGS synthesizes CβGs through the four enzymatic reactions: (i) initiation (transferring glucose to CGS), (ii) elongation of 1,2-β-glucan, (iii) regulation of DP of 1,2-β-glucan and (iv) cyclization [35]. The GH94 domain is involved in (iii) by controlling the length of the polysaccharide chain produced by the GT domain [18]. The GT domain is able to produce 1,2-β-glucan from UDP-glucose without the GH94 domain. Thus, CGS is virtually a glycosyl transferase, and the GH94 domain is enzymologically just an accessory domain. The phosphorolytic activity of the C-terminal domain (1493–2867) of CGS on linearized CβG was 0.725 U/mg [18], whereas the phosphorolytic activity of Lin1839r on Sop_3_ was much higher (19 s^−1^ = 9.2 U/mg). Therefore, Lin1839r is a novel enzyme that should be given a new EC number, and we propose 1,2-β-oligoglucan: phosphate α-glucosyltransferase as the systematic name and 1,2-β-oligoglucan phosphorylase as the short name for this Lin1839 protein. Sophorodextrin phosphorylase is a possible alternative name.

### Synthesis of 1,2-β-glucan and Sop_n_s

Lin1839r synthesized a series of Sop_n_s from Sop_2_ and Glc1*P* (Fig. 2C). It also produced 1,2-β-glucan from glucose and Glc1*P* without any detectable oligosaccharides at the early stage of the reaction, and then a series of Sop_n_s accumulated (Fig. 2B). These phenomena can be explained as described below.

**Figure 6:**
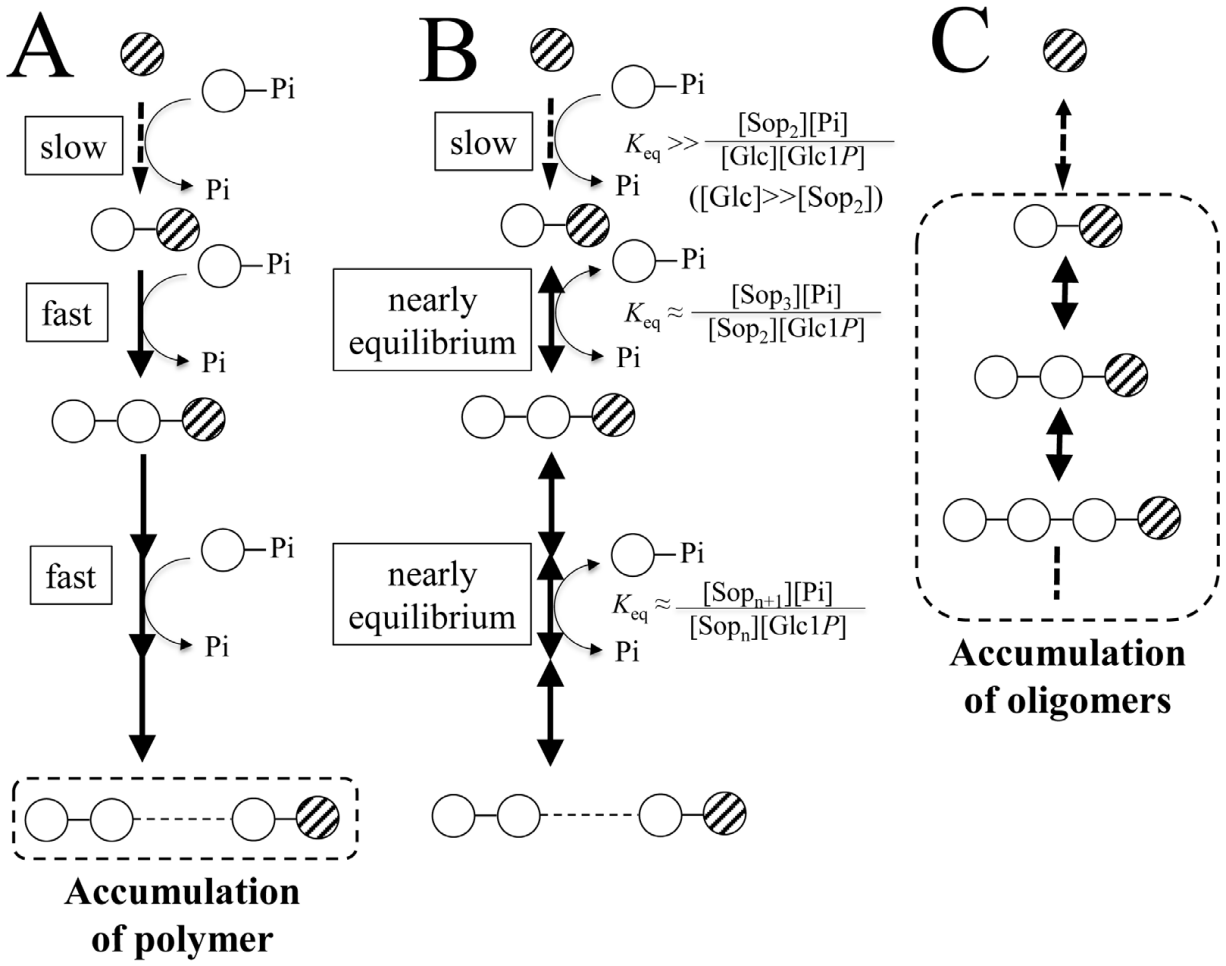
Possible mechanism of the production of 1,2-β-glucan and Sop_n_s from glucose and Glc1*P*. Possible reaction schemes at the beginning of the reaction (A), after Glc1*P*/Pi ratio reached equilibrium (B), and at the end of the reaction (C) are shown. Open and slashed circles represent the glucose moiety derived from Glc1*P* and material glucose, respectively. Lines connecting these circles represent the 1,2-β-glucosyl linkage. Dashed and bold arrows represent reactions with poor and preferential substrates, respectively. Glc1*P* is drawn with an open circle, and Pi represents inorganic phosphate. Accumulation of 1,2-β-glucan and Sop_n_s are surrounded by a dashed rounded box.

At the initial stage, glucose acted as a poor acceptor to generate Sop_2_ slowly. The resultant Sop_2_ was preferentially converted into a polymer because the Sop_2_ and Sop_n_s generated act as actual acceptors much more than glucose (Fig. 6A). Similar generation of polymeric compounds with a monosaccharide as the acceptor has been reported on production of cellulose-like material using cellodextrin phosphorylase [36]. The polymer formation continued until Glc1*P*/Pi ratio reached nearly equilibrium. Equilibriums between Sop_n_ and Sop_n+1_ (n≥2) were nearly completed at this stage (Fig. 6B). However, most part of glucose still remained in the reaction mixture, meaning that equilibrium between glucose and Sop_2_ was not completed (Fig. 6B). Sop_2_ was still being generated from glucose and Glc1*P*. The continuous formation of Sop_2_ increased the number of the Sop_n_ molecules, causing the decrease in the average DP of Sop_n_s. Finally, small oligosaccharides were concomitantly generated with disappearance of the polymer (Fig. 6C). This finding is in clear contrast to the reaction of cellodextrin phosphorylase in which the products immediately precipitated no longer participated in the reaction [36].

### Physiological Role of 1,2-β-oligoglucan Phosphorylase from *L. innocua*

Most β-1,2-linked glucose polymers have been found as CβG, a homopolymer of β-1,2-linked glucose with DP 17–24 [37]. CβG is distributed in some α-proteobacteria [38]–[40]. After CβG is synthesized by CGS, it is transported into periplasm by an ABC-transporter system involving Cgt protein. CβG is then modified with anionic molecules, such as succinate, in the case of *Brucella*. *Rhizobium phaseoli* secretes CβG as an exopolysaccharide [41]. CβG is involved in adaption to hypo-osmotic conditions for *Rhizobium meliloti* and *Agrobacterium tumefaciens* [42]. Defects in the *cgs* genes of these bacteria cause non-motile phenotypes because of defects in the assembly of flagella [43], [44]. *Brucella* CβG acts in lipid rafts found on host cell membranes to avoid the innate immune system [45]. CβG suppresses plant immune responses in the case of *Xanthomonas campestris* pv. *campestris* [46]. However, *L. inncoua* does not have a CGS homolog or synthetic system of CβG according to genomic information [47], implying that *L. inncoua* utilizes exogenous 1,2-β-glucan. A gene cluster is formed around the *lin1839* gene. A putative ATP-binding protein ABC-type transporter gene (*lin1841*–*lin1843*) may be involved in intake of Sop_n_s and/or 1,2-β-glucan. A putative GH3 β-glucosidase gene (*lin1840*) is thought to degrade the substrates with concerted action of the Lin1839 protein. LacI transcription factor gene (*lin1838*) may regulate expression of the genes in the gene cluster. Therefore, it is speculated that the gene cluster is involved in metabolism of Sop_n_s and/or 1,2-β-glucan in a specific environmental condition.

## Conclusion

Although numerous studies on 1,3- and 1,4-β-glucans have been reported, 1,2-β-glucan has not been further studied. This is probably because of the difficulty in obtaining 1,2-β-glucan and its oligomers. For instance, Sop_2_ is only available as an expensive reagent, although it has been well known since 1962 as a powerful inducer of cellulase from *Trichoderma reesei*, the hyper-cellulase-producing fungus [48]. We identified the Lin1839 protein as a novel 1,2-β-oligoglucan phosphorylase requiring a new EC number. This enzyme enables easy preparation of 1,2-β-glucan and its oligomers, including Sop_2_, and is an important milestone in the development of procedures for study of the functions of 1,2-β-glucan and related compounds.

## Supporting Information

Figure S1 NMR spectra of Sop_3_. (A) ^1^H-NMR, (B) ^13^C-NMR, (C) DQF-COSY, (D) TOCSY, (E) HSQC and (F) HMBC. I, II, and III denote first, second, and third glucose residues from reducing end, respectively. Letters in parenthesis represent position of hydroxyl group on the anomeric carbon. Arabic numbers shown with roman numbers represent positions of carbons and protons in sugar rings.
(PDF)

Figure S2 NMR spectra of Sop_4_. (A) ^1^H-NMR, (B) ^13^C-NMR, (C) DQF-COSY, (D) TOCSY, (E) HSQC and (F) HMBC. I, II, and III denote first, second, and third glucose residues from reducing end, respectively. Letters in parenthesis represent position of hydroxyl group on the anomeric carbon. Arabic numbers shown with roman numbers represent positions of carbons and protons in sugar rings.
(PDF)

Figure S3 NMR spectra of 1,2-β-glucan. (A) ^1^H-NMR, (B) ^13^C-NMR. Numbers under chemical shifts and in parenthesis represent positions of protons (A) and carbons (B). Letters in parenthesis represent position of hydroxyl group on the anomeric carbon.
(PDF)

Table S1 Chemical shifts in ^13^C-NMR and ^1^H-NMR spectra of Sop_3_.
(PDF)

Table S2 Chemical shifts in ^13^C-NMR and ^1^H-NMR spectra of Sop_4_.
(PDF)

Table S3 Chemical shifts in ^13^C-NMR and ^1^H-NMR spectra of 1,2-β-glucan.
(PDF)
