# Supplementary material for: 1,2-β-Oligoglucan Phosphorylase from Listeria innocua
Source: PLoS One. 2014 Mar 19;9(3):e92353. doi: 10.1371/journal.pone.0092353 (PMC3960220; doi:10.1371/journal.pone.0092353)
Supplement: Figure S1 — NMR spectra of Sop3. (A) 1H-NMR, (B) 13C-NMR, (C) DQF-COSY, (D) TOCSY, (E) HSQC and (F) HMBC. I, II, and III denote first, second, and third glucose residues from reducing end, respectively. Letters in parenthesis represent position of hydroxyl group on the anomeric carbon. Arabic numbers shown with roman numbers represent positions of carbons and protons in sugar rings. (PDF) [file pone.0092353.s001.pdf]

Figure S1. NMR specta of Sop_3.

(A) ^{1}H-NMR, (B) ^{13}C-NMR, (C) DQF-COSY, (D) TOCSY, (E) HSQC and (F) HMBC. I, II, and III denote first, second, and third glucose residues from reducing end, respectively. Letters in parenthesis represent position of hydroxyl group on the anomeric carbon. Arabic numbers shown with roman numbers represent positions of carbons and protons in sugar rings.

(A) ^{1}H 1D

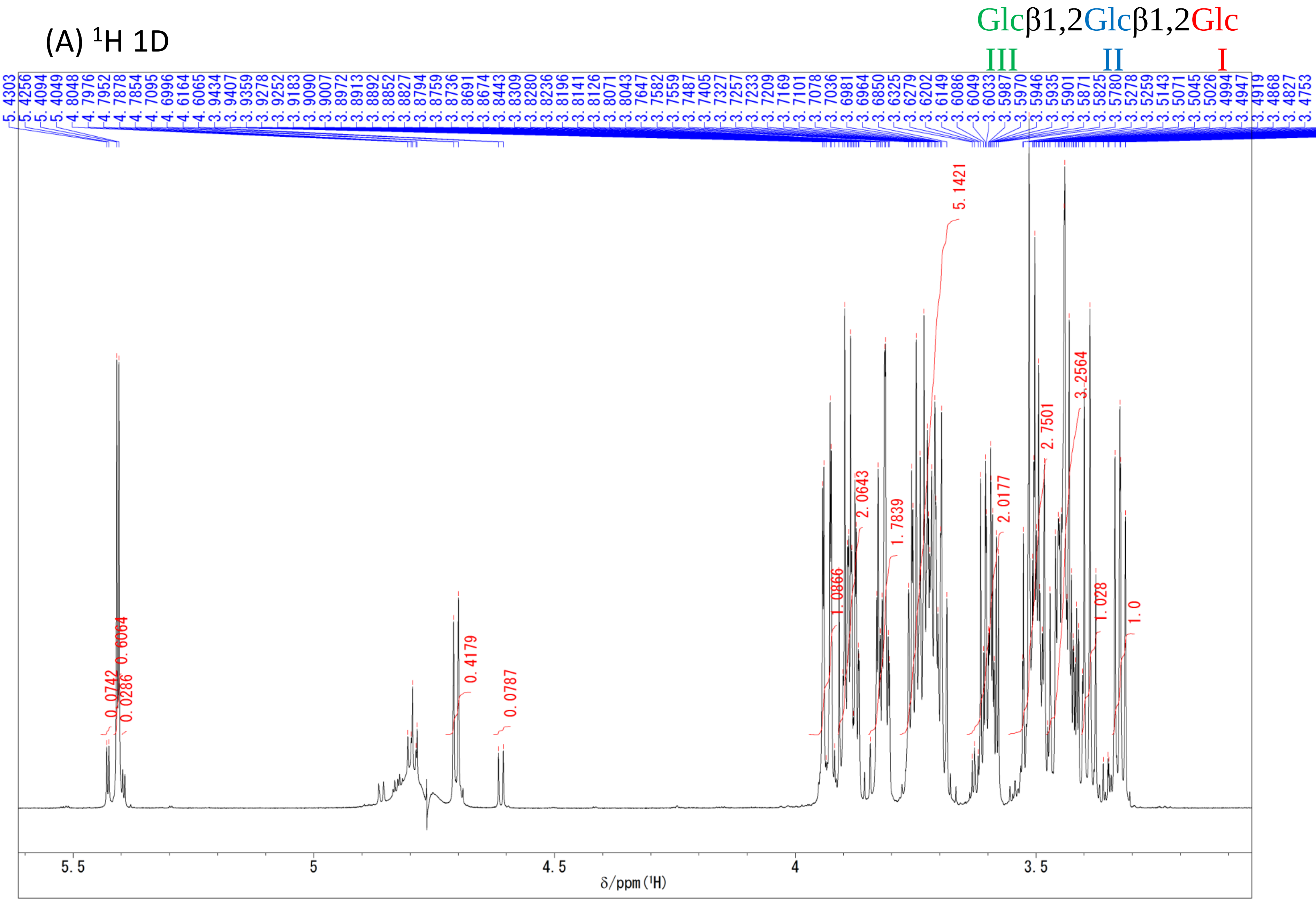

(B) ^{13}C 1D

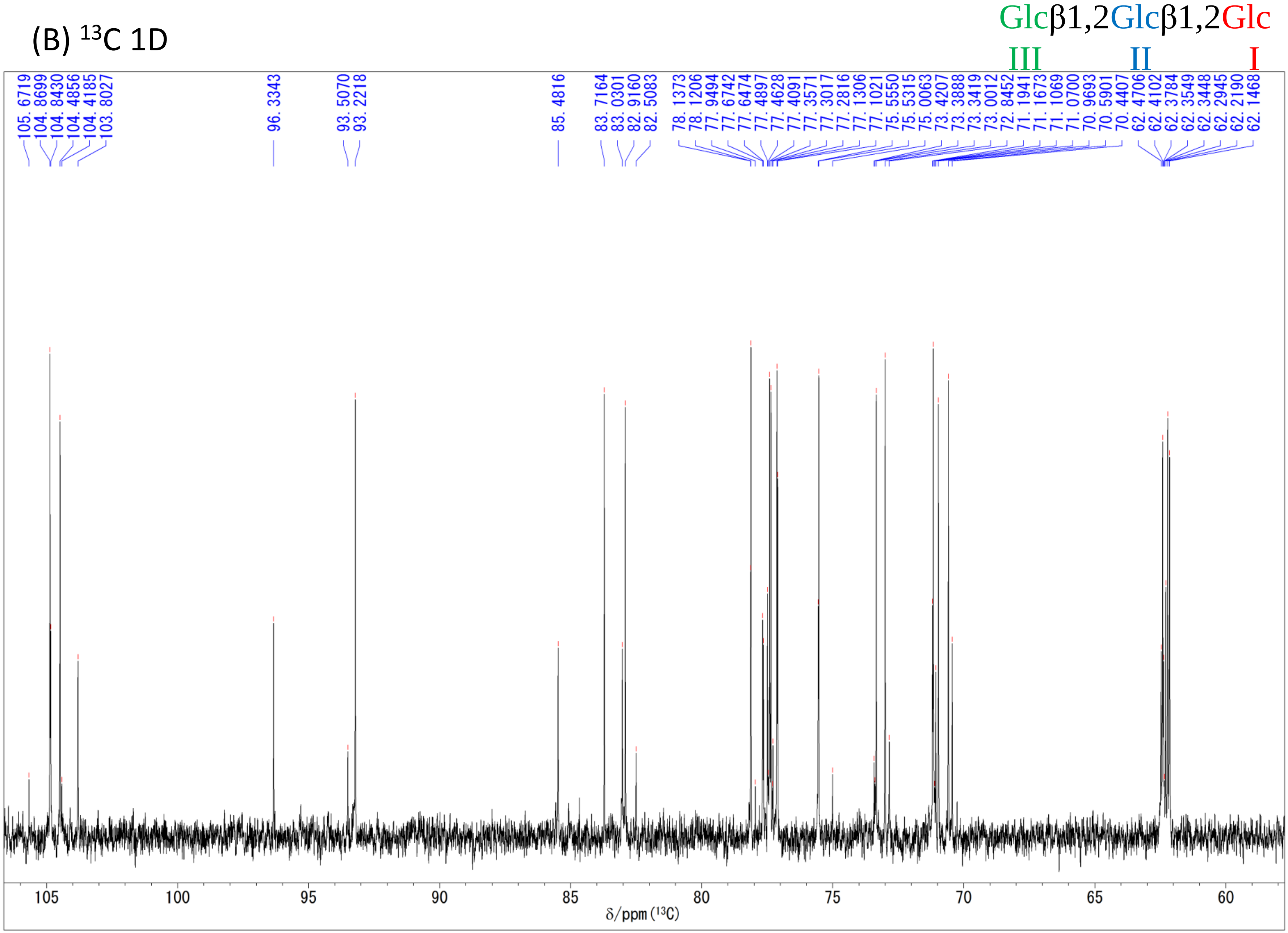
Glcβ1,2Glcβ1,2Glc
III
II
I
105. 6719
104. 8699
104. 8430
104. 4856
104. 4185
103. 8027
96. 3343
93. 5070
93. 2218
85. 4816
83. 7164
83. 0301
82. 9160
82. 5083
78. 1373
78. 1206
77. 9494
77. 6742
77. 6474
77. 4897
77. 4628
77. 4091
77. 3571
77. 3017
77. 2816
77. 1306
77. 1021
75. 5550
75. 5315
75. 0063
73. 4207
73. 3888
73. 3419
73. 0012
72. 8452
71. 1941
71. 1673
71. 1069
71. 0700
70. 9693
70. 5901
70. 4407
62. 4706
62. 4102
62. 3784
62. 3549
62. 3448
62. 2945
62. 2190
62. 1468
105
100
95
90
85
80
75
70
65
60
δ/ppm (13C)

(C) DQF-COSY

Glcβ1,2Glcβ1,2Glc

III II I

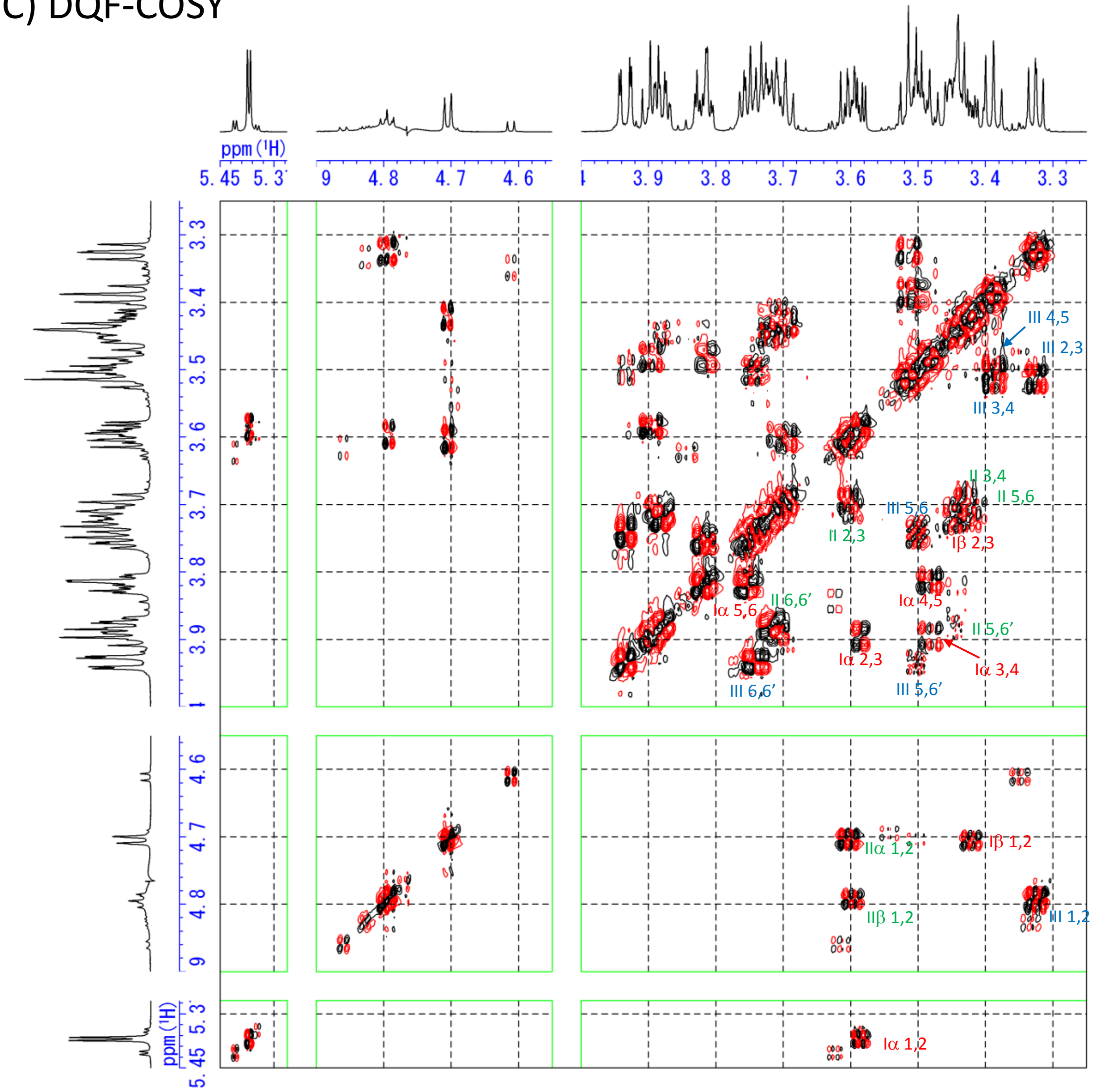

(D) TOCSY

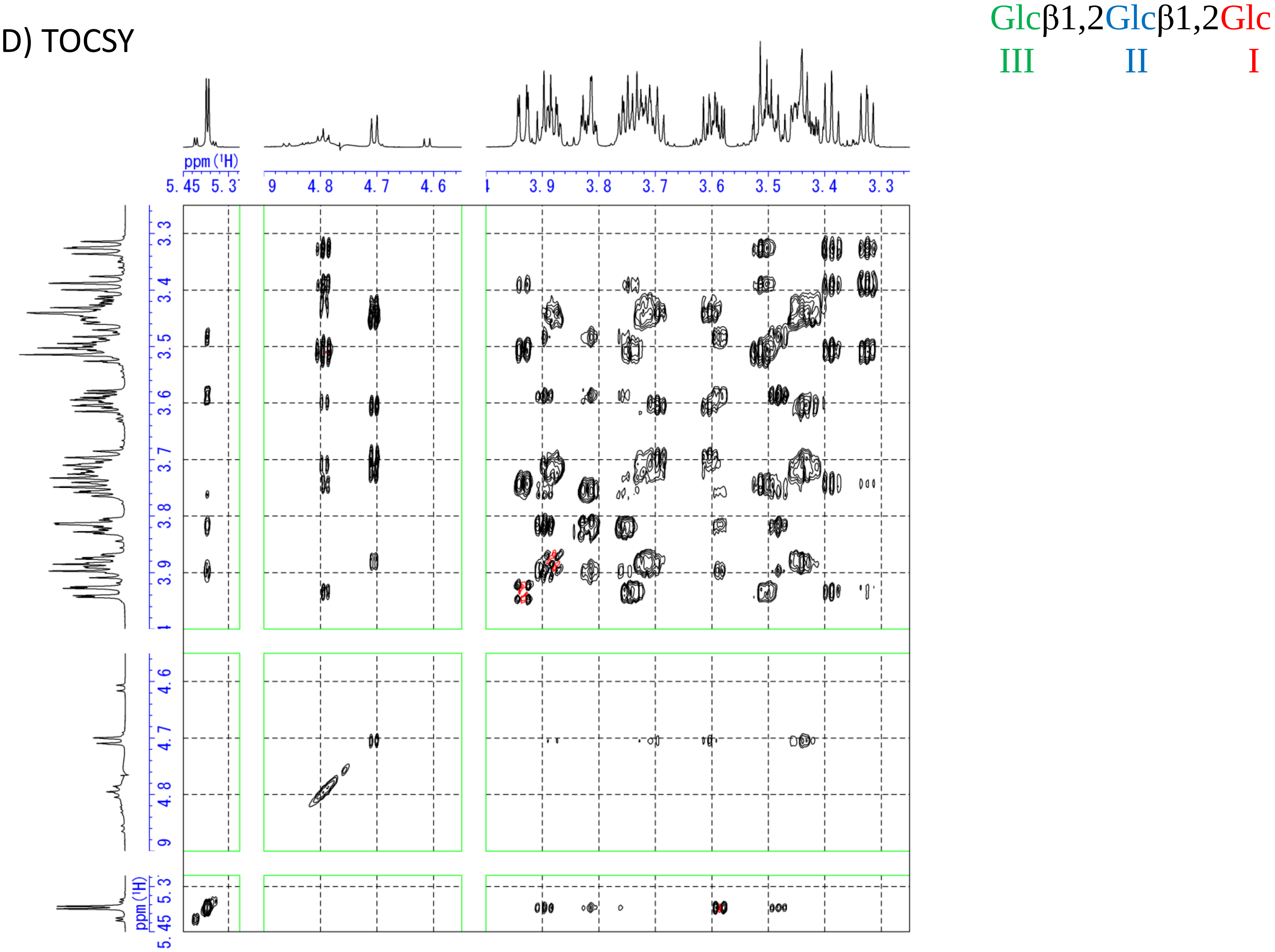

Glcβ1,2Glcβ1,2Glc
III II I
ppm (¹H)
5.45 5.3
4.9 4.8 4.7 4.6
4 3.9 3.8 3.7 3.6 3.5 3.4 3.3

(E) HSQC

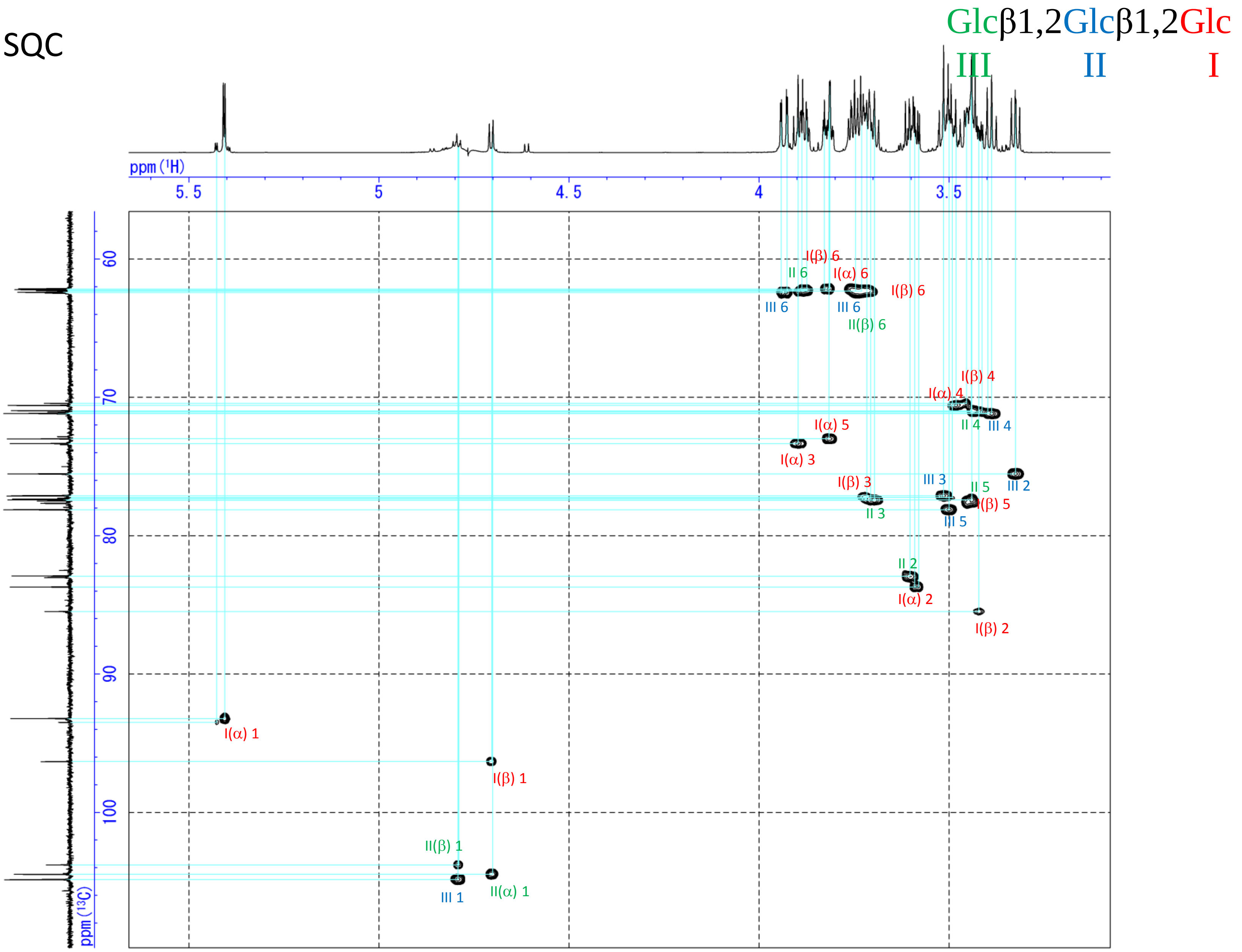

Glcβ1,2Glcβ1,2Glc
III
II
I
ppm (¹H)
5.5
5
4.5
4
3.5
ppm (¹³C)
60
70
80
90
100
I(β) 6
II 6
I(α) 6
I(β) 6
III 6
III 6
II(β) 6
I(β) 4
I(α) 4
II 4
III 4
I(α) 5
I(α) 3
I(β) 3
III 3
II 5
III 2
I(β) 5
II 3
III 5
II 2
I(α) 2
I(β) 2
I(α) 1
I(β) 1
II(β) 1
III 1
II(α) 1

(F) HMBC

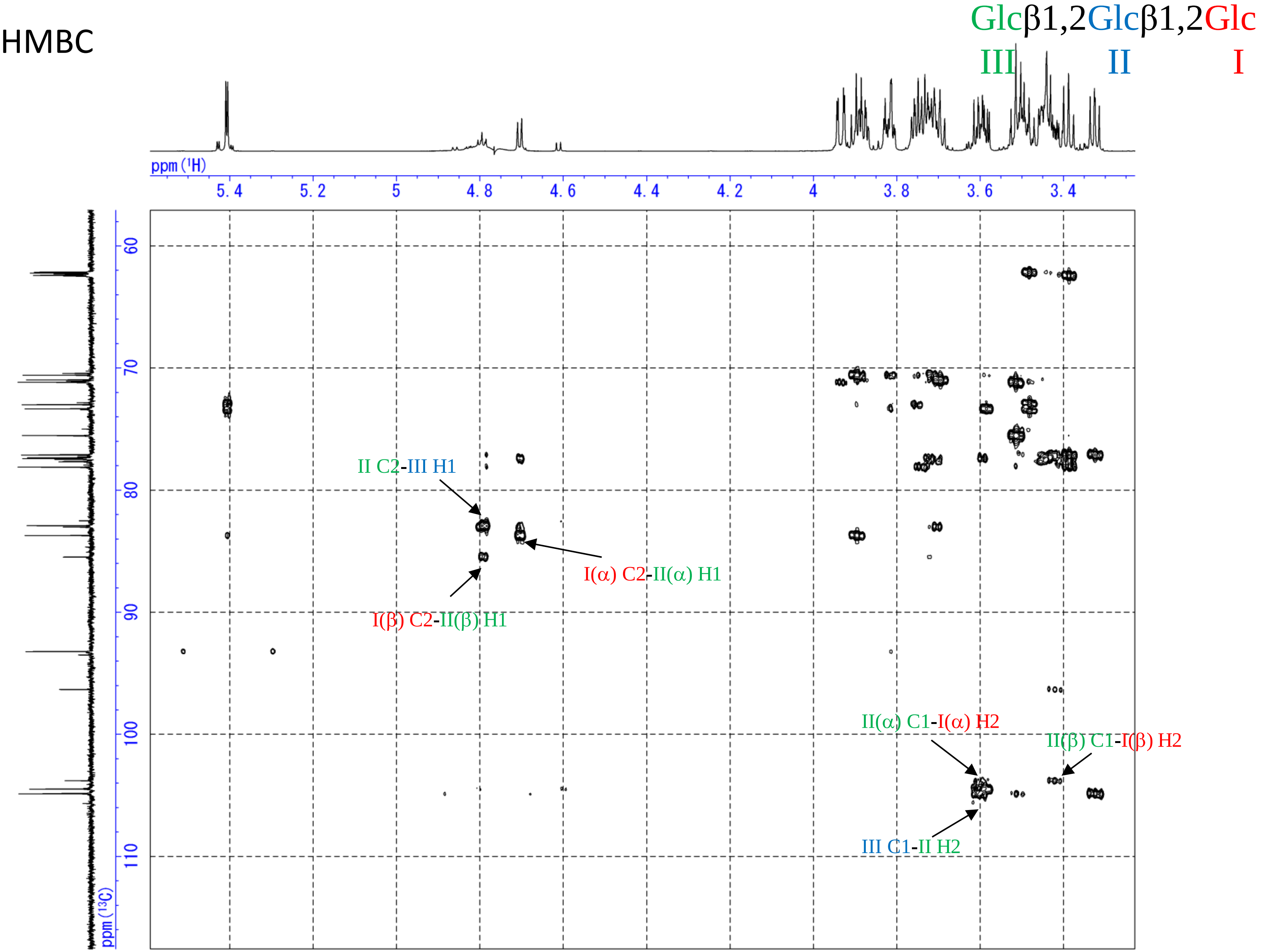
Glcβ1,2Glcβ1,2Glc
III
II
I
ppm (¹H)
5.4
5.2
5
4.8
4.6
4.4
4.2
4
3.8
3.6
3.4
ppm (¹³C)
60
70
80
90
100
110
II C2-III H1
I(α) C2-II(α) H1
I(β) C2-II(β) H1
II(α) C1-I(α) H2
II(β) C1-I(β) H2
III C1-II H2
