# Supplementary material for: 1,2-β-Oligoglucan Phosphorylase from Listeria innocua
Source: PLoS One. 2014 Mar 19;9(3):e92353. doi: 10.1371/journal.pone.0092353 (PMC3960220; doi:10.1371/journal.pone.0092353)
Supplement: Figure S2 — NMR spectra of Sop4. (A) 1H-NMR, (B) 13C-NMR, (C) DQF-COSY, (D) TOCSY, (E) HSQC and (F) HMBC. I, II, and III denote first, second, and third glucose residues from reducing end, respectively. Letters in parenthesis represent position of hydroxyl group on the anomeric carbon. Arabic numbers shown with roman numbers represent positions of carbons and protons in sugar rings. (PDF) [file pone.0092353.s002.pdf]

Figure S2. NMR spectra of Sop$_4$.

(A) ^{1}H-NMR, (B) ^{13}C-NMR, (C) DQF-COSY, (D) TOCSY, (E) HSQC and (E) HMBC. I, II, III, and IV denote first, second, and third glucose residues from reducing end, respectively. Letters in parenthesis represent position of hydroxyl group on the anomeric carbon. Arabic numbers shown with roman numbers represent positions of carbons and protons in sugar rings.

(A) ¹H 1D

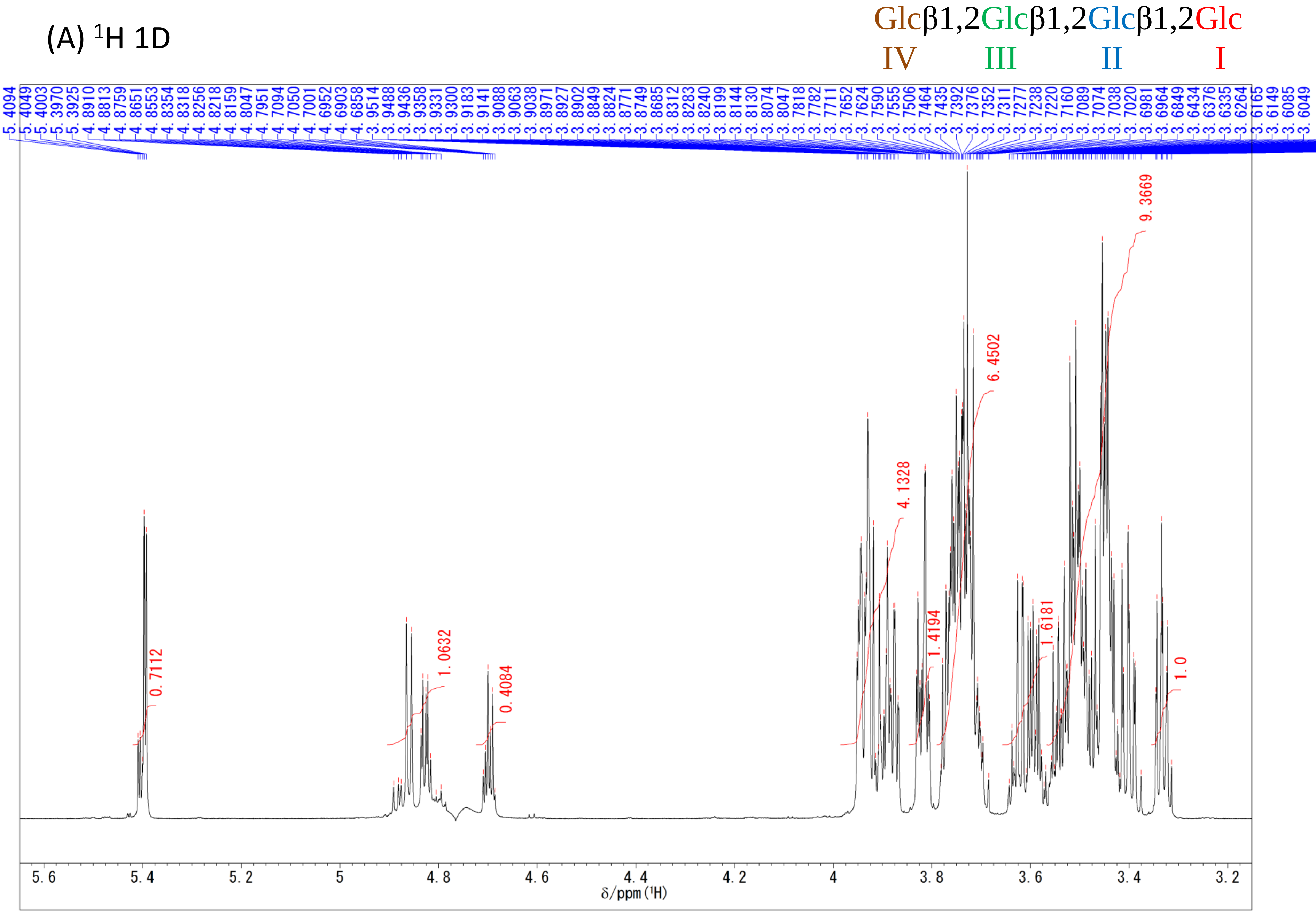

Glcβ1,2Glcβ1,2Glcβ1,2Glc
IV
III
II
I
5.4094
5.4049
5.4003
5.3970
5.3925
4.8910
4.8813
4.8759
4.8651
4.8553
4.8354
4.8318
4.8256
4.8218
4.8159
4.8047
4.7951
4.7094
4.7050
4.7001
4.6952
4.6903
4.6858
3.9514
3.9488
3.9436
3.9358
3.9331
3.9300
3.9183
3.9141
3.9088
3.9063
3.9038
3.8971
3.8927
3.8902
3.8849
3.8824
3.8771
3.8749
3.8685
3.8312
3.8283
3.8240
3.8199
3.8144
3.8130
3.8074
3.8047
3.7818
3.7782
3.7711
3.7652
3.7624
3.7590
3.7555
3.7506
3.7464
3.7435
3.7392
3.7376
3.7352
3.7311
3.7277
3.7238
3.7220
3.7160
3.7089
3.7074
3.7038
3.7020
3.6981
3.6964
3.6849
3.6434
3.6376
3.6335
3.6264
3.6165
3.6149
3.6085
3.6049
0.7112
1.0632
0.4084
4.1328
1.4194
6.4502
1.6181
9.3669
1.0
5.6
5.4
5.2
5
4.8
4.6
4.4
4.2
4
3.8
3.6
3.4
3.2
δ/ppm (¹H)

(B) ^{13}C 1D

Glcβ1,2Glcβ1,2Glcβ1,2Glc

IV III II I

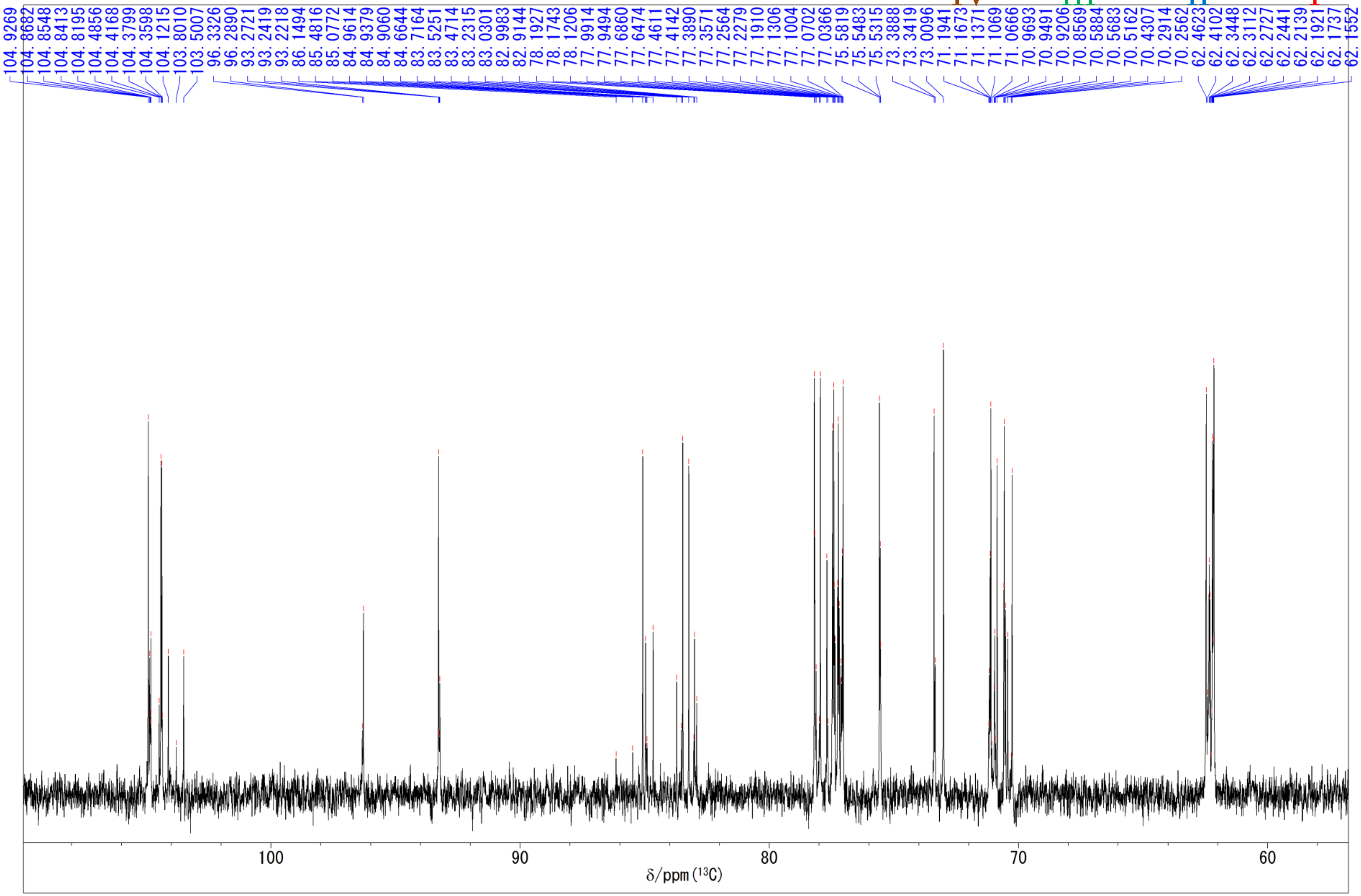

(C) DQF-COSY

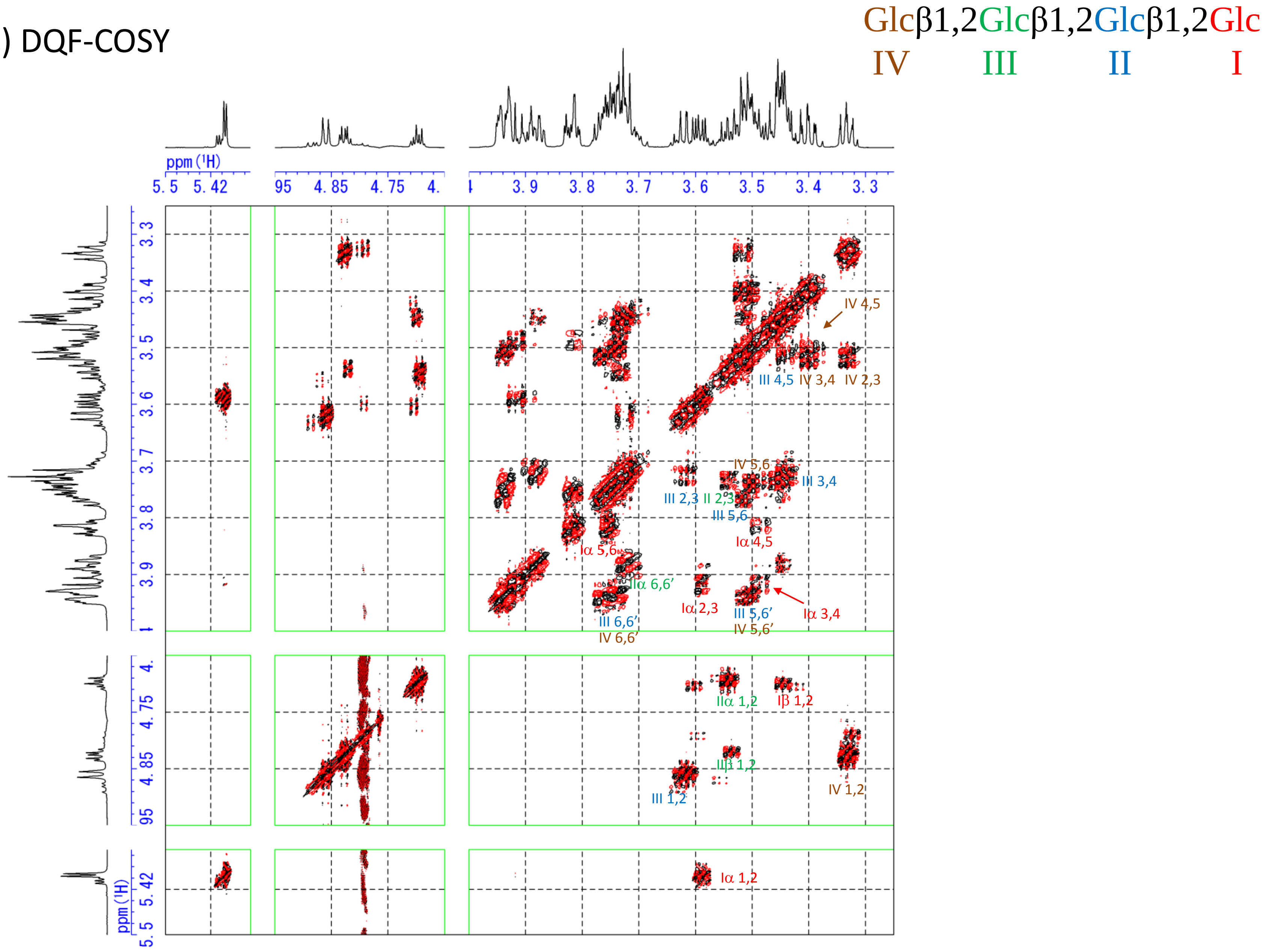

Glcβ1,2Glcβ1,2Glcβ1,2Glc
IV III II I
ppm (¹H)
IV 4,5
III 4,5 IV 3,4 IV 2,3
IV 5,6
III 3,4
III 2,3 II 2,3
III 5,6
Iα 4,5
Iα 5,6
IIα 6,6'
Iα 2,3
III 5,6'
IV 5,6'
Iα 3,4
III 6,6'
IV 6,6'
IIα 1,2
Iβ 1,2
IIβ 1,2
III 1,2
IV 1,2
Iα 1,2

(D) TOCSY

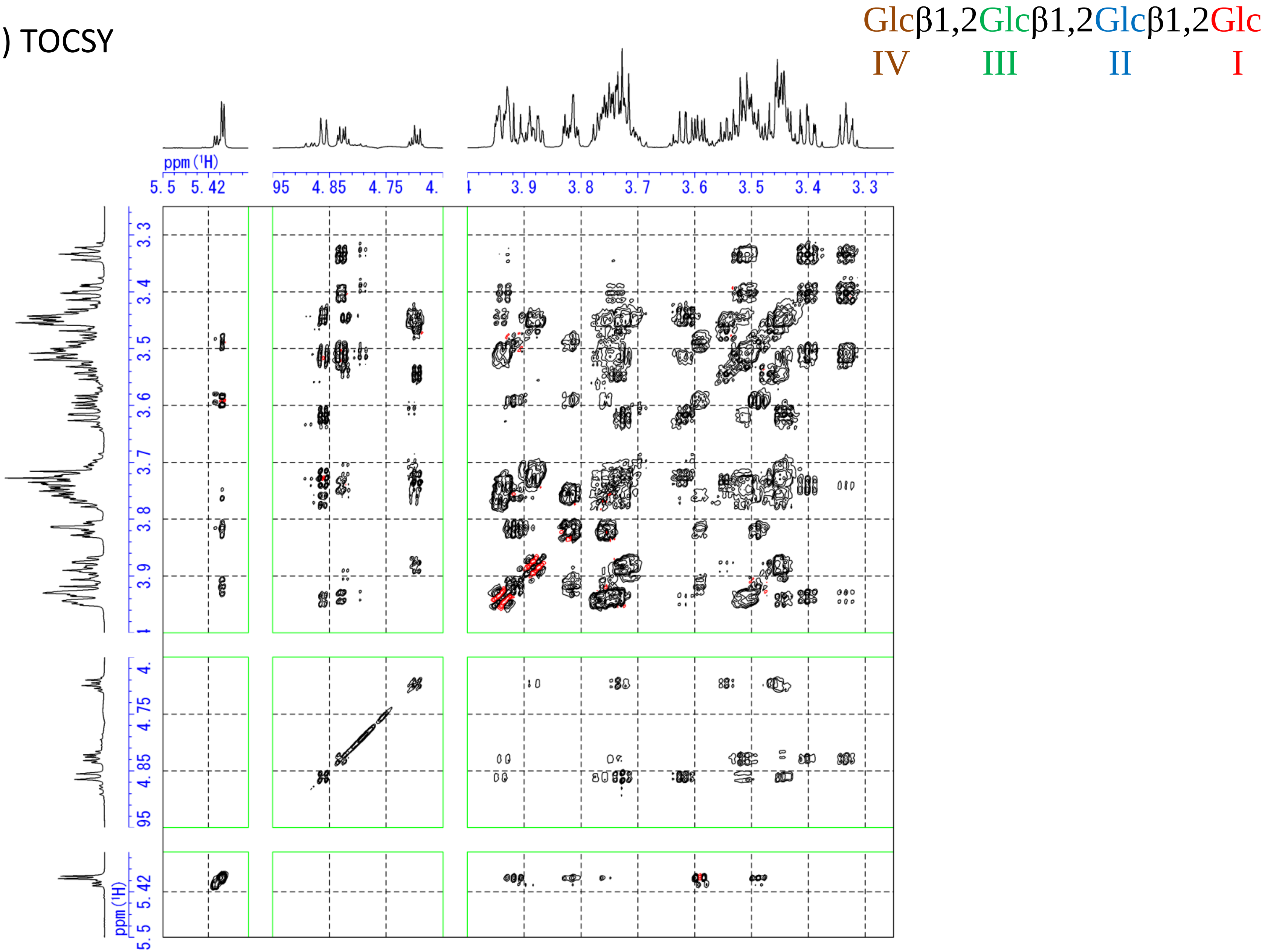

Glcβ1,2Glcβ1,2Glcβ1,2Glc
IV III II I
ppm (¹H)
5.5 5.42
95 4.85 4.75 4.
3.9 3.8 3.7 3.6 3.5 3.4 3.3

(E) HSQC

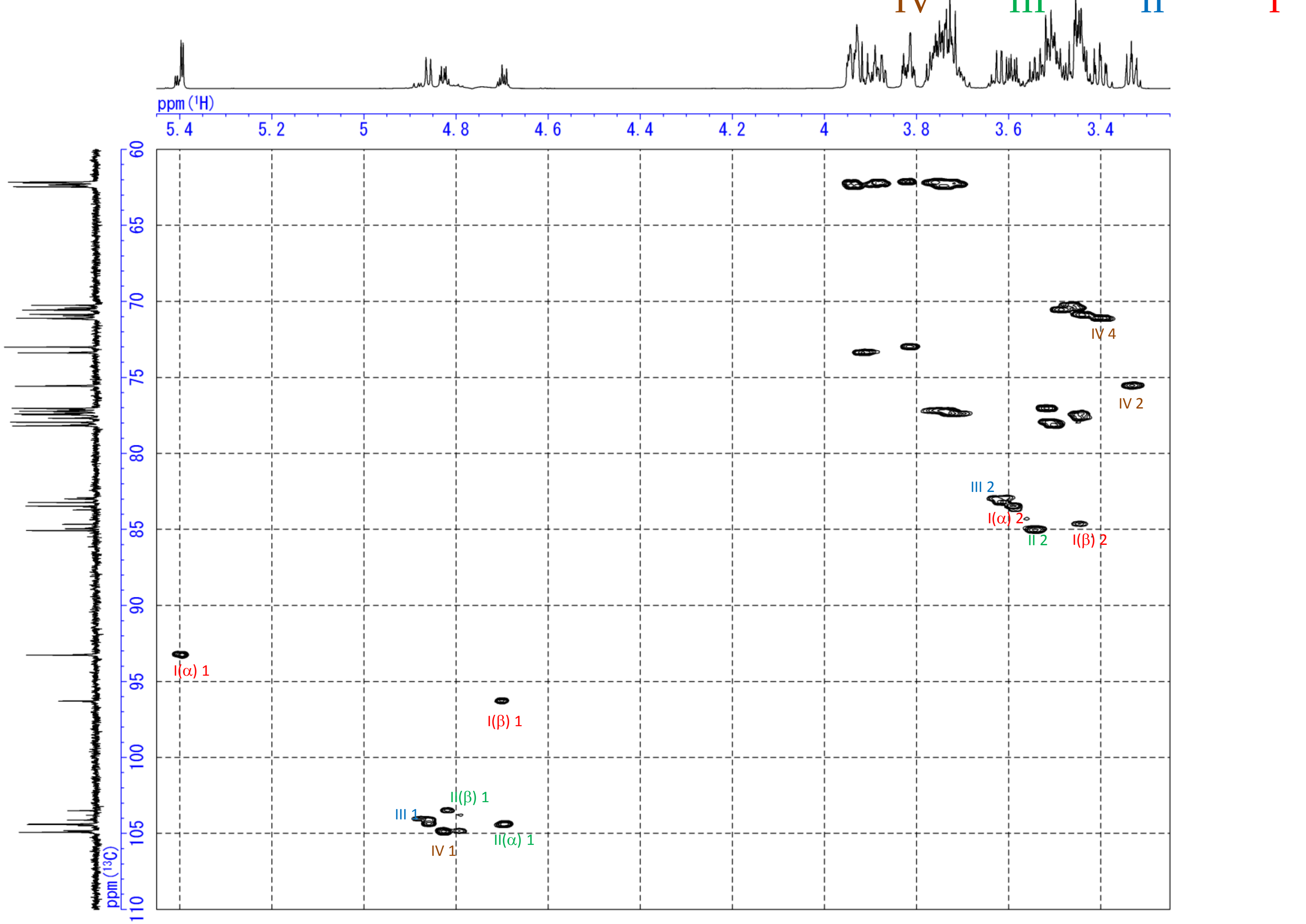

Glcβ1,2Glcβ1,2Glcβ1,2Glc
IV
III
II
I
ppm (¹H)
5.4
5.2
5
4.8
4.6
4.4
4.2
4
3.8
3.6
3.4
ppm (¹³C)
60
65
70
75
80
85
90
95
100
105
110
IV 4
IV 2
III 2
I(α) 2
II 2
I(β) 2
I(α) 1
I(β) 1
II(β) 1
III 1
II(α) 1
IV 1

(F) HMBC

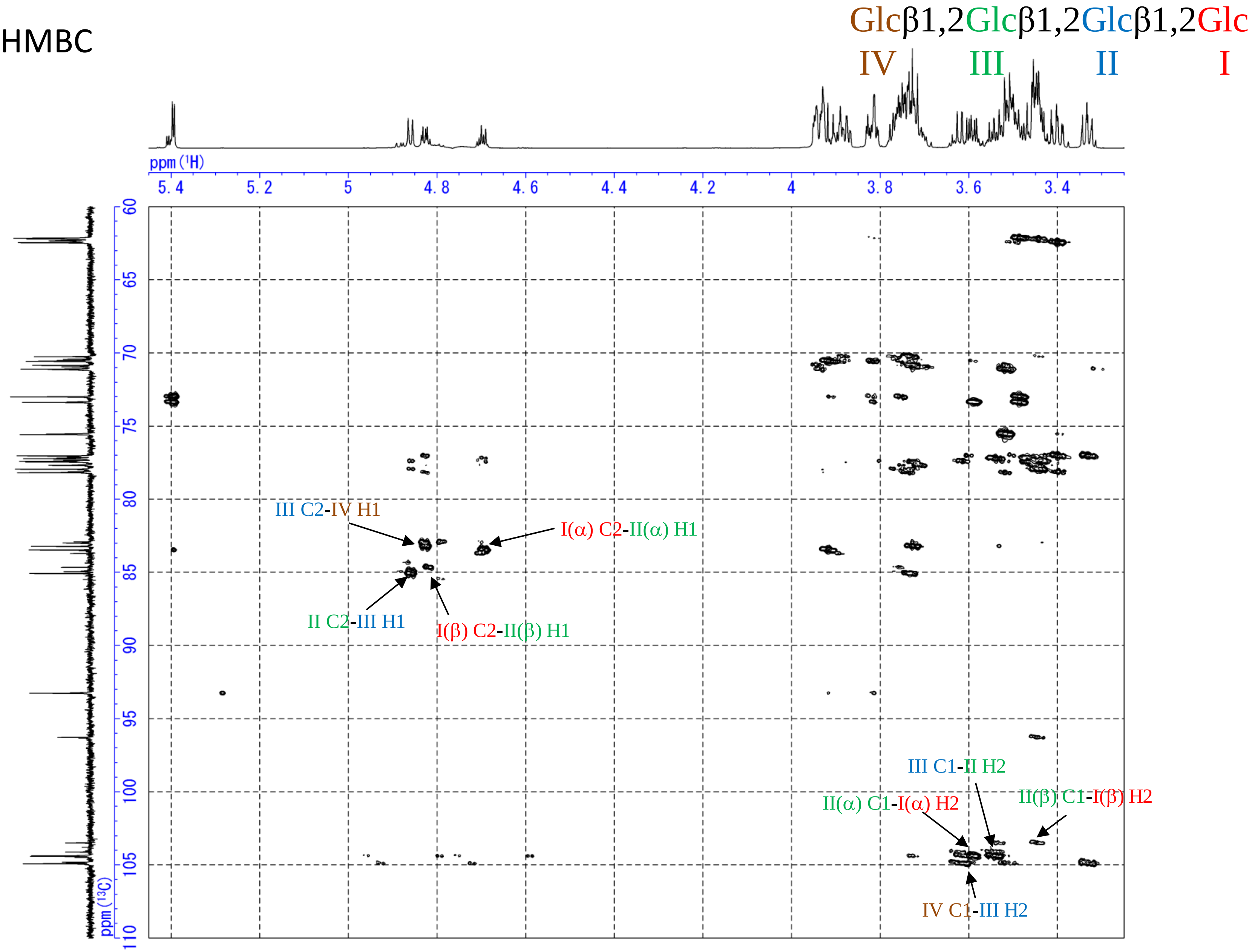

Glcβ1,2Glcβ1,2Glcβ1,2Glc
IV III II I
ppm (1H)
5.4 5.2 5 4.8 4.6 4.4 4.2 4 3.8 3.6 3.4
ppm (13C)
60 65 70 75 80 85 90 95 100 105 110
III C2-IV H1
I(α) C2-II(α) H1
II C2-III H1
I(β) C2-II(β) H1
III C1-II H2
II(α) C1-I(α) H2
II(β) C1-I(β) H2
IV C1-III H2
