# Supplementary material for: 1,2-β-Oligoglucan Phosphorylase from Listeria innocua
Source: PLoS One. 2014 Mar 19;9(3):e92353. doi: 10.1371/journal.pone.0092353 (PMC3960220; doi:10.1371/journal.pone.0092353)
Supplement: Figure S3 — NMR spectra of 1,2-β-glucan. (A) 1H-NMR, (B) 13C-NMR. Numbers under chemical shifts and in parenthesis represent positions of protons (A) and carbons (B). Letters in parenthesis represent position of hydroxyl group on the anomeric carbon. (PDF) [file pone.0092353.s003.pdf]

Figure S3. NMR spectra of 1,2-β-glucan.

(A) ^{1}H-NMR, (B) ^{13}C-NMR. Numbers under chemical shifts and in parenthesis represent positions of protons (A) and carbons (B). Letters in parenthesis represent position of hydroxyl group on the anomeric carbon.

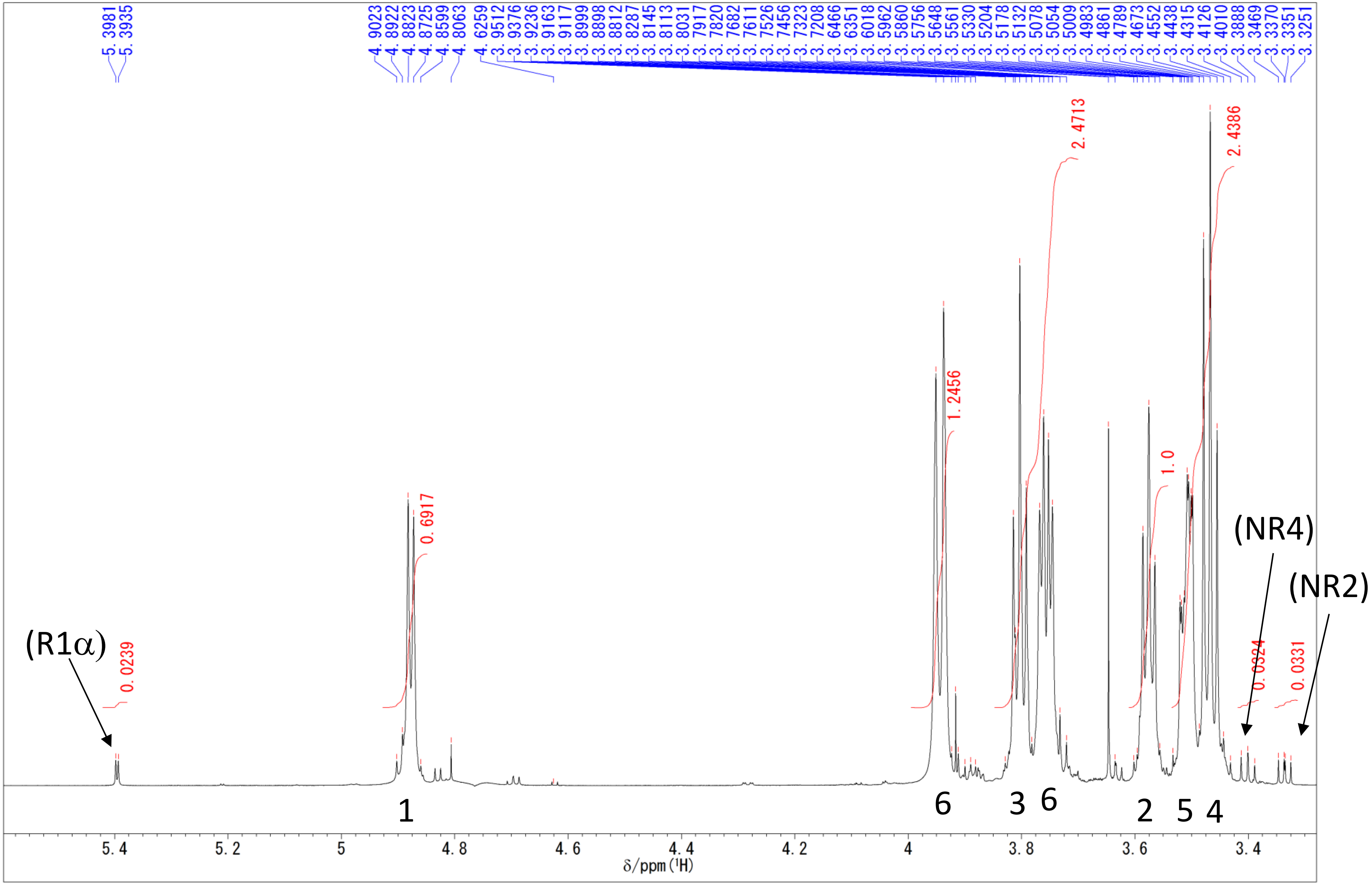

5.3981
5.3935
4.9023
4.8922
4.8823
4.8725
4.8599
4.8063
4.6259
3.9512
3.9376
3.9236
3.9163
3.9117
3.8999
3.8898
3.8812
3.8287
3.8145
3.8113
3.8031
3.7917
3.7820
3.7682
3.7611
3.7526
3.7456
3.7323
3.7208
3.6466
3.6351
3.6018
3.5962
3.5860
3.5756
3.5648
3.5561
3.5330
3.5204
3.5178
3.5132
3.5078
3.5054
3.5009
3.4983
3.4861
3.4789
3.4673
3.4552
3.4438
3.4315
3.4126
3.4010
3.3888
3.3469
3.3370
3.3351
3.3251
0.0239
0.6917
1.2456
2.4713
1.0
2.4386
0.0324
0.0331
(R1α)
(NR4)
(NR2)
1
6
3
6
2
5
4
5.4
5.2
5
4.8
4.6
4.4
4.2
4
3.8
3.6
3.4
δ/ppm (1H)

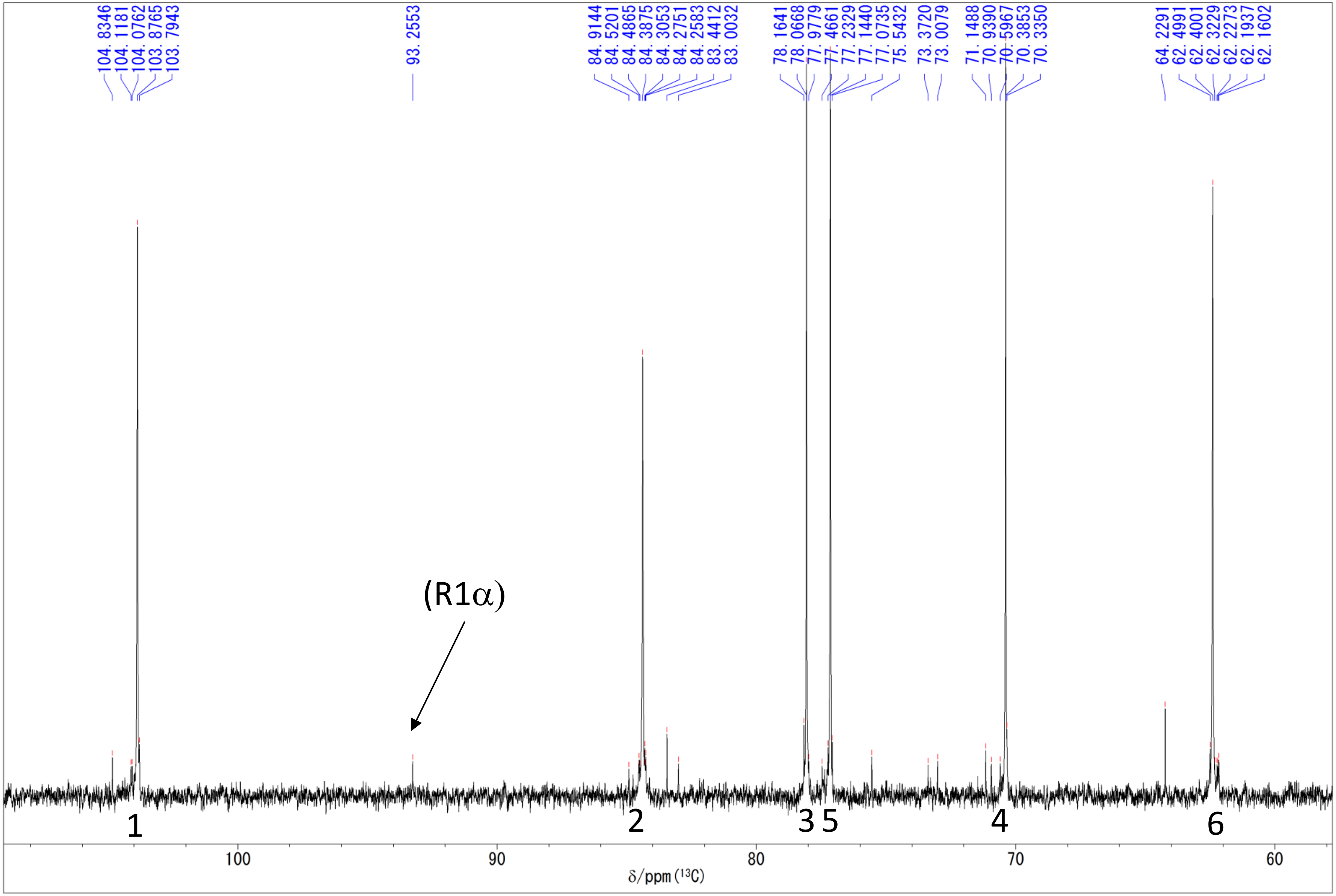
104. 8346
104. 1181
104. 0762
103. 8765
103. 7943
93. 2553
84. 9144
84. 5201
84. 4865
84. 3875
84. 3053
84. 2751
84. 2583
83. 4412
83. 0032
78. 1641
78. 0668
77. 9779
77. 4661
77. 2329
77. 1440
77. 0735
75. 5432
73. 3720
73. 0079
71. 1488
70. 9390
70. 5967
70. 3853
70. 3350
64. 2291
62. 4991
62. 4001
62. 3229
62. 2273
62. 1937
62. 1602
(R1α)
1
2
3
5
4
6
100
90
80
70
60
δ/ppm (13C)
