# Supplementary material for: 1,2-β-Oligoglucan Phosphorylase from Listeria innocua
Source: PLoS One. 2014 Mar 19;9(3):e92353. doi: 10.1371/journal.pone.0092353 (PMC3960220; doi:10.1371/journal.pone.0092353)
Supplement: Table S1 — Chemical shifts in 13C-NMR and 1H-NMR spectra of Sop3. (PDF) [file pone.0092353.s004.pdf]

Table S1. Chemical shifts in ^{13}C-NMR and ^{1}H-NMR spectra of Sop_3.

Sugar ring[a]	Position	α				β			
		^{13}C-NMR (δ ppm)	^{1}H-NMR (δ ppm)		*J* (Hz)	^{13}C-NMR (δ ppm)	^{1}H-NMR (δ ppm)		*J* (Hz)
I	1	93.2	5.41	d	$J_{1,2}$=3.6	96.3	4.70	d	$J_{1,2}$=7.9
	2	83.7[b]	3.59[c]	dd	$J_{2,3}$=9.7	85.5[d]	3.41-3.43[e]	m	
	3	73.3	3.88-3.91	m		77.1-7[h]	3.70-3.74	m	
	4	70.6	3.47-3.50	m		70.4	3.40-3.46	m	
	5	73.0	3.80-3.83	m		77.1-7[h]	3.42-3.47	m	
	6	62.1-5[h]	3.73-3.77	m		62.1-5[h]	3.70-3.77	m	
	6’						3.80-3.84	m	
II	1	104.5[c]	4.70[b]	d	$J_{1,2}$=7.9	103.8[e]	4.79[d]	d	$J_{1,2}$=7.8
	2	83.0[f]	3.59-3.62[g]	m	$J_{2,3}$=9.2	83.0	3.58-3.61	m	
	3	77.1-7[h]	3.68-3.72	m					
	4	71.2	3.42-3.47	m					
	5	77.1-7[h]	3.42-3.47	m					
	6	62.1-5[h]	3.70-3.74	m					
	6’		3.86-3.90	m					
III	1	104.9[g]	4.79[f]	d	$J_{1,2}$=8.0				
	2	75.5	3.32	dd	$J_{2,3}$=9.4				
	3	77.1-7[h]	3.50-3.53	m	$J_{3,4}$=9.2				
	4	71.0	3.39	dd	$J_{4,5}$=9.8				
	5	78.1	3.50	m					
					$J_{5,6'}$=2.1				
	6	62.1-5[h]	3.72-3.76	m	$J_{6,6'}$=12.4				
	6’		3.93	dd					

[a] I, II, and III denote first, second, and third glucoside residues from reducing end, respectively.

[b-g] represent HMBC correlations between the anomeric carbons of non-reducing end and the protons of reducing end, and between the anomeric protons of non-reducing end and the carbons of reducing end, respectively.

[h] represents that any one of chemical shifts between the value shown is assigned.

The signals are described as d = doublet; dd = doublet of doublet; m = multiplet.
