# Supplementary material for: 1,2-β-Oligoglucan Phosphorylase from Listeria innocua
Source: PLoS One. 2014 Mar 19;9(3):e92353. doi: 10.1371/journal.pone.0092353 (PMC3960220; doi:10.1371/journal.pone.0092353)
Supplement: Table S2 — Chemical shifts in 13C-NMR and 1H-NMR spectra of Sop4. (PDF) [file pone.0092353.s005.pdf]

Table S2. Chemical shifts in ^{13}C-NMR and ^{1}H-NMR spectra of Sop_4.

Sugar ring[a]	Position	α ^{13}C-NMR (δ ppm)	α ^{1}H-NMR (δ ppm)		α J (Hz)	β ^{13}C-NMR (δ ppm)	β ^{1}H-NMR (δ ppm)		β J (Hz)
I	1	93.3	5.39	d	$J_{1,2}$=3.6	96.2	4.70	d	$J_{1,2}$=7.8
	2	83.4[b]	3.59[c]	dd	$J_{2,3}$=9.7	84.7[d]	3.43-3.46[e]	m	
	3	73.4	3.90-3.93	m					
	4	70.6	3.47-3.50	m					
	5	73.0	3.80-3.83	m					
	6	62.2-5[j]	3.74-3.77	m					
	6’								
II	1	104.4[c]	4.70[b]	d	$J_{1,2}$=7.9	103.5[e]	4.83[d]	d	$J_{1,2}$=7.9
	2	85.1[f]	3.53-3.56[g]	m		85.0	3.52-3.55	m	
	3	77.0-5[j]	3.72-3.74	m					
	4	70.3	3.43-3.49	m					
	5								
	6	62.2-5[j]	3.70-3.74	m					
	6’		3.86-3.91	m					
III	1	104.4[g]	4.86[f]	d	$J_{1,2}$=7.9				
	2	83.2[h]	3.62[i]	dd	$J_{2,3}$=9.2				
	3	77.0-5[j]	3.71-3.74	m					
	4	70.9	3.43-3.46	m					
	5	77.0-3[j]	3.50-3.53	m					
	6	62.2-5[j]	3.75-3.78	m					
	6’		3.93-3.95	m					
IV	1	104.9[i]	4.83[h]	d	$J_{1,2}$=7.8				
	2	75.6	3.33	dd	$J_{2,3}$=9.5				
	3	77.0-3[j]	3.50-3.53	m					
	4	71.1	3.38-3.41	m					
	5	77.5-8.0[j]	3.49-3.52	m					
	6	62.2-5[j]	3.72-3.76	m					
	6’		3.92-3.95	m					

[a] I, II, III, and IV denote first, second, third, and fourth glucoside residues from reducing end, respectively.

[b-i] represent HMBC correlations between the anomeric carbons of non-reducing end and the protons of reducing end, and between the anomeric protons of non-reducing end and the carbons of reducing end, respectively.

[j] represents that any one of chemical shifts between the value shown is assigned.

The signals are described as d = doublet; dd = doublet of doublet; m = multiplet.
