# Supplementary material for: 1,2-β-Oligoglucan Phosphorylase from Listeria innocua
Source: PLoS One. 2014 Mar 19;9(3):e92353. doi: 10.1371/journal.pone.0092353 (PMC3960220; doi:10.1371/journal.pone.0092353)
Supplement: Table S3 — Chemical shifts in 13C-NMR and 1H-NMR spectra of 1,2-β-glucan. (PDF) [file pone.0092353.s006.pdf]

Table S3. Chemical shifts in ^{13}C-NMR and ^{1}H-NMR spectra of 1,2-β-glucan.

Sugar ring	Position	^{13}C-NMR (δ ppm)	^{1}H-NMR (δ ppm)		*J* (Hz)
Internal residue	1	103.9	4.88	d	$J_{1,2}$=7.8
	2	84.4	3.58	dd	$J_{2,3}$=9.1
	3	78.1	3.80	dd	$J_{3,4}$=9.1
	4	70.4	3.47	dd	$J_{4,5}$=10.0
	5	77.1	3.51	m	$J_{5,6}$=5.7
	6	62.4	3.76	dd	$J_{6,6'}$=12.4
	6’		3.94	m	
Reducing end	1(α)	93.2	5.40		
Non-reducing end	2		3.34		
	4		3.40		

The signals are described as d = doublet; dd = doublet of doublet.
